# Comorbidities and Concomitant Medications in Middle-Aged Japanese People According to the Charlson Comorbidity Index and Age: Results of the NDB-K7Ps-Study-3

**DOI:** 10.3390/epidemiologia7020034

**Published:** 2026-03-02

**Authors:** Airi Sekine, Kei Nakajima

**Affiliations:** 1 Department of Nutritional Science, Faculty of Food and Nutritional Sciences, Japan Women’s University, Tokyo 112-8681, Japan; sekinea@fc.jwu.ac.jp; 2 Department of Endocrinology and Diabetes, Saitama Medical Center, Saitama Medical University, Kawagoe 350-8550, Japan

**Keywords:** national database, diagnosis, prescribed medication, health checkups, Charlson Comorbidity Index

## Abstract

**Background/Objectives**: The Charlson Comorbidity Index (CCI), which focuses on 19 comorbid diseases and conditions, has been widely used as a valid predictor of mortality. This study aimed to comprehensively examine the prevalence of nearly all comorbidities and concomitant medications according to CCI classification (CCI = 0 and CCI ≥ 4) and age group (aged 40–44 and 70–74 years) in middle-aged Japanese adults. **Methods**: The present study included 9,182,226 individuals who underwent health checkups from April 2018 to March 2019. A total of 15,916 cases of diagnosed diseases and conditions, including communicable diseases; diseases of the eye, ear, skin, and musculoskeletal system; and psychiatric disorders, were investigated alongside 16,886 prescribed medications. **Results**: The prevalence of allergic rhinitis was ranked among the leading comorbidities in all age groups and CCI categories. Individuals with a CCI ≥ 4 in the 40–44 age group showed a higher prevalence of cardiometabolic diseases such as hypertension, diabetes, and dyslipidemia compared with individuals with a CCI = 0 in the 70–74 age group. Furthermore, individuals with a CCI ≥ 4 in the 40–44 age group also had a higher prevalence of communicable diseases, gastrointestinal symptoms, iron deficiency anemia, and psychiatric disorders compared with individuals with a CCI = 0 in the 70–74 age group. The ranking for prescribed medications was essentially the same between age groups, but was found to differ between CCI categories. **Conclusions**: This study identified overlooked comorbidities and concomitant medications that are not accounted for in the 19 conditions included in the CCI, which may be important prognostic factors in determining mortality. Although patients with more comorbidities were found to be more frequently diagnosed with cardiometabolic diseases regardless of their age, the presence of pharmacotherapy may be dependent on age.

## 1. Introduction

The Charlson Comorbidity Index (CCI), developed by Charlson et al. in 1987, is widely used to determine the survival rate in patients with multiple comorbidities based on 19 diseases and conditions [1,2]. The CCI has been considered a confounding factor in outcome studies for predicting patient prognoses when adjusting for potential disease severity [3,4].

Because the CCI was specifically designed to quantify comorbidity burden in relation to mortality risk, rather than to capture the overall prevalence of all comorbidities, previous CCI-based studies have been limited to the few specific diseases included in the CCI. Therefore, little is known about the comprehensive prevalence of diagnosed diseases and prescribed medications that are not included among the criteria of the CCI, such as dyslipidemia, communicable diseases, eye and ear diseases, dermatological and musculoskeletal diseases, and psychiatric disorders. Particularly, psychiatric disorders and allergic diseases have been reported to be associated with additional increased mortality and adverse health outcomes in population-based studies [5,6,7]. Comprehensively investigating the relationship between the CCI and comorbidities that are not among the 19 comorbidities in the CCI may provide valuable and novel insights for disease prevention and health promotion in future epidemiological research (Figure S1).

The risk of cardiometabolic diseases, type 2 diabetes, and metabolic syndrome increases markedly from middle age (40 years and older) [8], and the onset of these conditions during this period has major implications for long-term morbidity, mortality, and healthcare costs. Middle-aged adults therefore represent a critical population for prevention and early lifestyle intervention. In Japan, all health insurers are required to provide health checkups for individuals aged 40–74 years and promote healthy lifestyle changes. The data from these checkups, along with nationwide health insurance claims, are integrated in the National Database of Health Insurance Claims and Specific Health Checkups (NDB). This database also contains comprehensive information on diagnosed diseases and prescribed medications, and covers nearly the entire Japanese population, making it a valuable resource for large-scale epidemiological studies.

Previous studies have developed algorithms to determine the CCI using claims databases such as the NDB, primarily relying on the International Classification of Diseases (9th and 10th Revisions (ICD-9 and ICD-10) codes) [9,10,11,12]. However, the original CCI was designed for medical chart data and includes criteria that are not discernable from ICD-10 codes; for example, included individuals with diet-controlled diabetes are excluded from the CCI criteria, which may lead to overestimation of comorbidity scores.

This study aimed to comprehensively examine the prevalence of diagnosed diseases and prescribed medications according to CCI classification and age group among Japanese adults aged 40–74 years, with a particular focus on age-related differences within CCI categories.

## 2. Methods

### 2.1. Study Design

This study was a cross-sectional and sub-study of the National Database Study in the Kanto 7 Prefectures (Tokyo, Kanagawa, Saitama, Chiba, Ibaraki, Gunma, and Tochigi; NDB-K7Ps Study), which aimed to investigate the clinical factors that are primarily associated with cardiometabolic diseases. The database comprises data on disease diagnoses, prescribed medications, and annual health checkup information for Japanese people aged 40–74 years [13]. The details of the NDB-K7Ps Study’s concept and design are provided in [14].

### 2.2. Data Source and Study Population

The present study included data on 10,183,619 individuals of both sexes who were living in any of the above seven prefectures of Kanto and who underwent specific health checkups from April 2018 to March 2019, which are mandatory for people aged 40–74 years in Japan [15]. The participation rate in the annual health checkup in the Kanto 7 Prefectures from April 2018 to March 2019 was 58.3% [16].

It was important to exclude individuals with implausibly low or implausibly high serum parameters, while minimizing the removal of clinically meaningful extreme values; therefore, we excluded data for individuals whose measurements were below the 0.01st percentile or above the 99.9th percentile for body mass index, systolic blood pressure (SBP), diastolic blood pressure (DBP), high-density lipoprotein cholesterol, low-density lipoprotein cholesterol (LDL-C), triglycerides, aspartate aminotransferase, alanine aminotransferase, or γ-glutamyl transferase (Table S1). This method for measuring the biochemical parameters was described in a previously published protocol [17]. After these data were excluded, data for a total of 9,182,226 individuals were analyzed in this study.

### 2.3. Hospitalization and Demographic Data

Demographic data were obtained from the health checkup data in the NDB. Individuals with severe conditions or disabilities requiring hospitalization or residence in a nursing home were unlikely to be included in this study because the NDB health checkup data cover individuals who are able to undergo health checkups at their workplace or through their local government.

### 2.4. Disease Diagnosis and Prescribed Medications

Japan’s disease codes are used for insurance claims in Japan [18] and they correspond to disease classifications which are more detailed than ICD-10 codes [19,20]. In this study, we identified 15,916 diseases using Japan’s disease codes, corresponding to categories from A to N in the ICD-10; namely, all diagnosed diseases and conditions except for maternal and neonatal diseases, poisoning, and accidental injury. In Japan, the diagnostic term “hyperlipidemia” was officially renamed to “dyslipidemia” in 2007; however, the previous term was still widely used in insurance claims data in 2018 [21]. In the present study, we retained the original diagnostic terms as recorded in the database to accurately reflect real-world clinical practice. Similarly, we identified 16,886 prescribed medications using Japan’s medication codes for insurance claims in Japan, excluding herbal medicine and diagnostic reagents. These codes correspond to a detailed classification of medications, namely the dosage, form, and method of administration (powder, tablet, capsules, injection, tape, or patch), trade name, whether it is an original or generic product, and the pharmaceutical company that manufactures the drug. For example, Acetaminophen (original product) is separately coded as “620002023” and “620000033” for doses of 200 and 300 mg, respectively, in Japan’s medication codes. Information about therapeutic category was collected from the package insert or the search system of the Prescription Medications in Pharmaceuticals and Medical Devices Agency [20]. The disease and medication codes on insurance claims are rigorously checked by medical clerks in each hospital, clinic, and dispensing pharmacy. We defined individuals with each diagnosis and medication as those who were diagnosed or prescribed medication at least once from April 2018 to March 2019 in order to comprehensively include both acute and chronic conditions.

### 2.5. Charlson Comorbidity Index

The Charlson Comorbidity Index (CCI), originally developed and validated by Charlson et al. [1], was calculated using a previously validated algorithm based on ICD-10 codes [9,22], with minor adaptations for the Japanese NDB. The Charlson Comorbidity Index (CCI) is calculated as the simple sum of the weighted index (1, 2, 3, or 6 points) of 19 comorbidities according to their potential influence on mortality; the CCI includes diabetes with/without diabetic complications, congestive heart failure, peripheral vascular disease, chronic pulmonary disease, mild and severe liver disease, hemiplegia, renal disease, leukemia, lymphoma, metastatic tumor, and AIDS [1]. In this study, we determined the CCI using both diagnosed diseases and conditions as well as prescribed medication from the Japanese database, the NDB. Detailed definitions of the 19 comorbidities based on the ICD-10 codes were selected by Quan and Ono [9,22], and other criteria (identified using Japan’s medication codes) are shown in Table S2. We defined individuals with “diabetes without chronic complications” as those diagnosed with at least 1 of 30 disease codes for diabetes without chronic complications and who were prescribed at least 1 of 487 oral hypoglycemic agents or 61 insulin preparations without insulin for intravenous injection (vial formulation). Therefore, individuals with diabetes who were not receiving pharmacotherapy (e.g., early and diet-controlled diabetes) were not included in the CCI. Individuals who had “diabetes with chronic complications” were defined as those with diabetic retinopathy, neuropathy, or nephropathy, or who were hospitalized with diabetic ketoacidosis (DKA) or hyperosmolar hyperglycemic syndrome (HHS). In this study, individuals with diabetes who were prescribed at least 1 of 23 medications for diabetic retinopathy were included in the group with diabetic retinopathy. We defined individuals with DKA or HHS as those who were diagnosed with DKA, HHS, or diabetic coma. We also defined hospitalized individuals with DKA or HHS as those who were prescribed an insulin preparation for intravenous injection. “Mild liver disease” refers to diagnoses of chronic hepatitis or cirrhosis without portal hypertension. “Moderate liver disease” includes diagnoses of chronic hepatitis or cirrhosis with portal hypertension, and “severe liver disease” includes diagnoses of variceal bleeding. Metastatic tumor includes diagnoses of end-stage cancer and unknown primary cancer. Several definitions in the CCI, such as hospitalization, detailed symptoms with electrocardiogram change, New York Heart Association functional classification, and serum creatinine are unavailable in this database; these are described in Table S2. For example, several common chronic conditions such as hypertension, dyslipidemia, and hyperuricemia or gout are not included in the CCI criteria. To disclose the difference most clearly in the prevalence of diagnosed diseases and prescribed medications between individuals with no comorbidities and those with a high burden of comorbidities, we categorized the study individuals into CCI = 0 and CCI ≥ 4. This approach was chosen to avoid heterogeneity, as the severity of diseases varies widely among individuals with moderate comorbidities (CCI = 1–3). Previous studies have defined CCI ≥ 4 as high comorbidity and have reported its association with mortality [23]. In contrast, CCI = 0 represents an appropriate reference group without major comorbidity. In this study, age-adjusted CCI was used, which is a highly important predictor of long-term mortality [1,24].

### 2.6. Statistical Analysis

The prevalence of all diagnosed diseases and prescribed medications is expressed as n (%) and was rank ordered. The clinical characteristics of the included individuals are expressed as mean ± standard deviation or median (interquartile range). To protect participant privacy, age data in the NDB are provided in 5-year categories; therefore, individual-level age data were unavailable in the NDB. To evaluate subject age as a numeric value, we transformed age groups into substitute ages corresponding to the median for each age group (42, 47, 52, 57, 62, 67, and 72 years, respectively). Statistical significance of difference between CCI = 0 and CCI ≥ 4 was compared using analysis of co-variance adjusting for S-age. To disclose the difference most clearly in the prevalence of diagnosed diseases and prescribed medications between younger and older adults, we categorized the study individuals into 40–44 and 70–74 years, representing the youngest and oldest available age groups in the database [25]. In this study, age-adjusted CCI was calculated as the simple sum of the weighted index as supplementary information to aid interpretation (aged 40–44: 1 or 70–74: 4 points; therefore, CCI ≥ 4 aged 40–44: age-adjusted CCI ≥ 5, CCI ≥ 4 aged 70–74: age-adjusted CCI ≥ 8), which is a highly important predictor of long-term mortality [1,24]. Continuous and categorical variables were analyzed using Student’s t-test and the χ^2^ test, respectively. Logistic regression models were used to examine the prevalence of each disease, comparing the group with a CCI ≥ 4 and aged 40–44 years to the group with a CCI = 0 and aged 70–74 years. The results are presented as odds ratios (ORs) with 95% confidence intervals (CIs). The models were adjusted for potential confounders: sex, BMI, SBP, DBP, triglycerides (TG), low-density lipoprotein cholesterol (LDL-C), high-density lipoprotein cholesterol (HDL-C), glycated hemoglobin (HbA_1c_), pharmacotherapy for hypertension, diabetes, and dyslipidemia, smoking status, habitual exercise (≥30 min per session, >2 times/week vs. less frequent), and habitual alcohol consumption and frequency. The χ^2^ test, to determine the prevalence of prescribed medications, was conducted using EZR version 1.68 (Jichi Medical University, Tochigi, Japan), which is a graphical user interface for R (The R Foundation for Statistical Computing, Vienna, Austria) [25]. Other statistical analyses were conducted using SAS-Enterprise Guide (SAS-EG 7.1) in SAS version 9.4 (SAS Institute, Cary, NC, USA). Two-tailed *p* < 0.05 was considered statistically significant. When differences in the proportion of patients who were diagnosed selected diseases among four groups (age and CCI categories) were evaluated by the χ^2^ test, values of *p* < 0.007 were considered to represent statistical significance on the basis of the Bonferroni test.

### 2.7. Ethics Approval of Research

The study protocol was approved by Japan’s Ministry of Health, Labour and Welfare (MHLW) in December 2020, and we received digitally recorded anonymous data in July 2022 (No. 2320). This study was carried out in accordance with the guidelines set out in the Declaration of Helsinki and was approved by the Institutional Review Board of the Ethics Committee of Japan Women’s University (No. 513; 11 May 2022). The details of the data processing that was conducted to link the health checkup data with the datasets, comprising diagnosed diseases and prescribed medications, were provided in the previous study [26]. Informed consent was not required because of the anonymous data of the MHLW as part of its nationwide program involving the provision of medical data to third parties [27]. On its website, The MHLW publicly announces the provision of the NDB data to researchers for secondary analyses [28]. In addition, our institution posts information regarding the use of these data on its website, including the purpose of use and responsible investigators [29]. The public can request details of the research results, and such requests are addressed individually. This process is in accordance with the “opt-out” provisions of the Japanese Ethical Guidelines for Medical and Biological Research Involving Human Subjects.

## 3. Results

Table 1 shows the 30 most-diagnosed diseases and conditions among all included individuals. Table S3 shows the 100 most-diagnosed diseases and conditions, or, specifically, those with over 1% prevalence. Allergic rhinitis was the most prevalent among all individuals, followed by hypertension, acute bronchitis, allergic conjunctivitis and astigmatism. In Table 2, the 30 most frequently prescribed medications are organized according to their therapeutic category, nonproprietary name, whether they are original or generic products, and their dosage. Table S4 shows the 50 most frequently prescribed medications in detail. Loxoprofen Sodium Hydrate (60 mg, generic) was the most frequently prescribed among all individuals, followed by Rebamipide (100 mg, generic), L-Carbocisteine (500 mg, generic), Tranexamic Acid (250 mg, generic) and Acetaminophen (200 mg, original). The therapeutic categories of medication ranking within the top 20 were antipyretics and analgesics, anti-inflammatory drugs, antitussives and allergic agents, expectorants, and common cold drugs. The most frequently prescribed medication for cardiometabolic disease was Rosuvastatin Calcium (2.5 mg, generic), a hyperlipidemic agent. The most frequently prescribed antidiabetic agents and antihypertensives were Metformin Hydrochloride (250 mg, original; n = 81,290; 0.80%) and azilsartan (20 mg, original; n = 68,157; 0.67%), respectively, which were among the top 50 most frequently prescribed medications (Table S4).

Table 3 shows the clinical characteristics of individuals according to their CCI category. The proportions of the study population with CCI = 0 and CCI ≥ 4 were 62.9% and 3.42% of all individuals, respectively. Individuals with a CCI ≥ 4 were found to be older, with higher body mass index, SBP, DBP, TG, aspartate aminotransferase, alanine aminotransferase, γ-glutamyl transferase, and glycated hemoglobin (HbA_1c_) levels and lower LDL-C levels in comparison with individuals with a CCI = 0 (all *p* < 0.0001). In all individuals, the most prevalent condition among the 19 CCI comorbidities was chronic pulmonary disease, followed by mild liver disease and peptic ulcer disease. In the CCI ≥ 4 group, the most prevalent disease among CCI comorbidities was mild liver disease, followed by chronic pulmonary disease and peptic ulcer disease. The most prevalent chronic pulmonary disease was asthmatic bronchitis both in all the included individuals (n = 919,088; 65.3% of individuals with chronic pulmonary disease) and in the individuals in the CCI ≥4 group (n = 86,782; 60.6% of individuals with chronic pulmonary disease). Similarly, the most prevalent peptic ulcer disease was gastric ulcer, both in all included individuals (n = 598,864; 82.1% of individuals with peptic ulcer disease) and in the individuals in the CCI ≥ 4 group (n = 118,679; 84.0% of individuals with peptic ulcer disease). The most prevalent mild liver disease among all included individuals was liver dysfunction (n = 357,731; 41.6% of individuals with mild liver disease), and the most prevalent mild liver disease in the CCI ≥ 4 group was fatty liver (n = 62,757; 42.9% of individuals with mild liver disease), followed by liver dysfunction (n = 58,466; 39.9% of individuals with mild liver disease). Among other diagnosed diseases, the proportions of hypertension and dyslipidemia were higher among the individuals in the CCI ≥ 4 group than those in the CCI = 0 group (all *p* < 0.0001).

Table 4 and Table 5 present the 30 most frequently diagnosed diseases and conditions, stratified by CCI category (0 or ≥4) and age group (40–44 or 70–74 years). Supplementary Tables S5–S8 provide extended rankings, including the 100 most frequently diagnosed diseases and conditions and those with a prevalence greater than 1%. Accordingly, key findings are summarized in Table 4 and Table 5, whereas detailed results are shown in Supplementary Tables S5–S8. In individuals with CCI = 0 and age 40–44 (Table 4), the three leading diseases were allergic rhinitis, acute bronchitis and astigmatism. Among cardiometabolic diseases, hypertension was ranked 22nd in the group with CCI = 0, aged 40–44. In individuals with CCI = 0, aged 70–74 (Table 4), the three most-diagnosed diseases were hypertension, allergic rhinitis and cataract. Among cardiometabolic diseases, hyperlipidemia was the 8th leading comorbidity and unspecified diabetes mellitus was ranked 22nd. In individuals with CCI ≥ 4, aged 40–44 (Table 5), the most prevalent diseases were allergic rhinitis, followed by hypertension, asthmatic bronchitis and unspecified diabetes mellitus. In individuals with CCI ≥ 4, aged 70–74 (Table 5), the most prevalent diseases were hypertension, followed by unspecified diabetes mellitus, allergic rhinitis and hyperlipidemia. Among both CCI groups, individuals aged 40–44 years had a higher prevalence of allergic rhinitis, asthmatic bronchitis, acute bronchitis, and acute upper respiratory infection than individuals aged 70–74 years (all *p* < 0.05 using Student’s *t*-test; Table 4 and Table 5). Moreover, individuals aged 70–74 years had a higher prevalence of hypertension, cataract, allergic conjunctivitis, hypermetropic astigmatism, astigmatism, chronic gastritis, hyperlipidemia, unspecified diabetes mellitus, and dyslipidemia than individuals aged 40–44 years (all *p* < 0.05 using Student’s *t*-test; Table 4 and Table 5). However, in groups with CCI ≥ 4, there were no significant differences between individuals aged 40–44 and those aged 70–74 years in the prevalence of allergic conjunctivitis or fatty liver.

Among both age groups, individuals in the CCI ≥ 4 group had a higher prevalence of all diseases than those in the CCI = 0 group (all *p* < 0.001 using Student’s *t*-test). Moreover, individuals with CCI ≥ 4 who were in the early 40s age group had a higher prevalence of nearly all diseases in comparison with individuals in the CCI = 0 group who were in their early 70s; this was with the exception of age-related diseases such as cataract, hypermetropic astigmatism, osteoporosis, gonarthrosis, and lumbar spondylosis deformans.

Notably, logistic regression analysis demonstrated that the CCI ≥ 4 and age 40–44 group had a higher prevalence of many diseases compared with the CCI = 0 and age 70–74 group (Table S22). Elevated risks were particularly pronounced for cardiometabolic diseases, including type 2 diabetes mellitus, hypertension, and dyslipidemia. In addition, increased odds were observed for gastrointestinal conditions (e.g., gastritis and constipation), psychiatric disorders (e.g., depressive episodes), and other conditions such as iron deficiency anemia and hypothyroidism. Table 6 shows the prevalence of diagnosed diseases stratified by CCI category (0 or ≥4) and age group (40–44 or 70–74 years). All differences between the CCI categories and the age groups were statistically significant using χ^2^ tests with Bonferroni correction for multiple comparisons. Individuals in the CCI ≥4, aged 40–44 group had a higher prevalence of all diseases compared with those in the CCI = 0, aged 70–74 group (all adjusted *p* < 0.007).

The prevalence of diagnosed diseases was expressed as n (% each group). All differences between age and CCI categories were significant (all *p* values < 0.007; according to χ^2^ test with Bonferroni correction for multiple comparisons).

Table 7 and Table 8 show the 30 most frequently prescribed medications, stratified by CCI category (0 or ≥4) and age group (40–44 or 70–74 years). Supplementary Tables S9–S12 provide detailed information on the 50 most frequently prescribed medications. Accordingly, key findings are summarized in Table 7 and Table 8, while extended rankings are presented in Supplementary Tables S9–S12. The three most frequently prescribed medications among included individuals aged 40–44 years were Loxoprofen Sodium Hydrate (60 mg, generic), Rebamipide (100 mg, generic), and L-Carbocisteine (500 mg, generic), in the CCI = 0 and CCI ≥ 4 groups. Similarly, the three most frequently prescribed medications among included individuals aged 70–74 years were Rebamipide (100 mg, generic), Loxoprofen Sodium Hydrate (60 mg, generic), and L-Carbocisteine (500 mg, generic), in both the CCI = 0 and CCI ≥ 4 groups. The most frequently prescribed medication for cardiometabolic disease was Rosuvastatin Calcium (2.5 mg generic); hyperlipidemic agents ranked among the 10 most frequently prescribed medications for included individuals aged 70–74 years in both the CCI = 0 and CCI ≥ 4 groups. Vitamin D (eldecalcitol; 0.75 µg, original) ranked among the 30 most frequently prescribed medications among the included individuals aged 70–74 years in both the CCI = 0 and CCI ≥ 4 groups. Vitamin B12 (mecobalamin; 500 µg, original) was ranked among the 30 most frequently prescribed medications among the individuals in both the 40–44 and 70–74 age groups with CCI ≥ 4, and only among individuals who were aged 70–74 in the CCI = 0 group. Among both CCI groups, individuals aged 40–44 years had a higher prevalence of being prescribed Loxoprofen Sodium Hydrate, Tranexamic Acid, L-Carbocisteine, Acetaminophen, Fexofenadine Hydrochloride, Dextromethorphan Hydrobromide Hydrate, and Cefcapene Pivoxil Hydrochloride Hydrate than individuals aged 70–74 years (all *p* < 0.05 using Student’s *t*-test; Table 7 and Table 8). Moreover, individuals aged 70–74 years had a higher prevalence of being prescribed Rebamipide, Rosuvastatin Calcium, and Loxoprofen Sodium Hydrate Tape/Patch than individuals aged 40–44 years (all *p* < 0.05 using Student’s *t*-test; Table 7 and Table 8). However, in the CCI ≥ 4 groups, there were no significant differences between individuals aged 40–44 and 70–74 years in the prevalence of Metformin Hydrochloride prescription.

Therapeutic categories were allocated according to the package insert or the Prescription Medications in Pharmaceuticals and Medical Devices Agency’s search system [20].

## 4. Discussion

In this study, we examined the prevalence of nearly all comorbidities and concomitant medications according to CCI scores and age groups among middle-aged Japanese individuals using a real-world administrative database. Within the same CCI categories, individuals aged 40–44 years were found to more frequently have allergic rhinitis and communicable diseases such as upper respiratory infections. In contrast, those aged 70–74 years more often had cardiometabolic and other chronic conditions (Table 4 and Table 5). However, logistic regression analysis further demonstrated that young individuals with more comorbidities had a higher prevalence of several conditions, including communicable diseases, musculoskeletal disorders, gastrointestinal symptoms, iron deficiency anemia, and psychiatric disorders, compared with older individuals with no comorbidities (Table S22). These results suggest that certain conditions may be associated with mortality risk, regardless of age. Particularly, iron deficiency anemia showed one of the strongest associations. Considering that iron deficiency anemia has been associated with increased mortality across multiple causes [30], this finding highlights a clinically meaningful comorbidity that may be overlooked when focusing on conventional CCI-defined conditions. These findings suggested that the CCI may reflect aging processes, particularly those increasing the prevalence of cardiometabolic diseases in younger individuals. Notably, there was a discrepancy between diagnosed diseases and prescribed medications in individuals aged 40–44 years with a CCI ≥ 4. For example, three antidiabetic agents—Sitagliptin Phosphate Hydrate, Linagliptin, and Metformin Hydrochloride—were among the 50 most frequently prescribed medications in older individuals with high comorbidities. However, these medications were not among the top 50 medications prescribed to younger individuals. Although the individuals with more comorbidities were more frequently diagnosed with cardiometabolic diseases regardless of age, whether or not they receive pharmacotherapy for their cardiometabolic diseases may be dependent on their age.

The predominance of anti-inflammatory and gastric medications across all groups is consistent with the high prevalence of related diagnosed conditions, such as allergic rhinitis and gastritis, observed in this study (Table 4 and Table 5). In addition, these medications are commonly prescribed in general internal medicine settings and can be initiated by non-specialists, which likely contributes to their high frequency across age and CCI categories [20]. Additionally, eldecalcitol (vitamin D) was ranked among the five most-prescribed medications only among women aged 70–74 years, which corresponds to a high prevalence of osteoporosis in this age group (Tables S16 and S20). Mecobalamin (vitamin B12), which is prescribed for peripheral neuropathy, neuralgia, and numbness, was ranked among the 30 most-prescribed medications (Table 6 and Table 7). This result was observed not only in older individuals but also in younger individuals with more comorbidities. This finding suggests that treatment with mecobalamin may be associated with greater disease severity and mortality.

This study identified several conditions (such as hypertension, iron deficiency anemia, hypothyroidism, and psychiatric disorders) that were found to be associated with potential mortality risk but which are not included in the CCI. These conditions are, however, accounted for in the Elixhauser Comorbidity Index [6] and the Chronic Disease Score [7,30,31,32], both of which reference a broader range of diseases than the CCI. Although these indices differ in design and purpose, and neither consistently outperforms the CCI in predicting mortality and disease severity [33,34,35,36], their inclusion of these conditions points to certain limitations of the CCI. In particular, sleep disorders, which were the most frequent psychiatric disorders in this study, are not recorded in any of these indices despite evidence linking them to increased all-cause and cardiovascular mortality [37,38,39]. These findings suggest that incorporating selected non-CCI conditions into comorbidity assessments may improve their ability to reflect disease severity and mortality risk; this warrants further investigation.

In this study, individuals with high comorbidities had a high prevalence of common communicable diseases, such as acute bronchitis and upper respiratory infection. Because these diseases can induce pneumonia, which is the leading cause of death, the relationship between mortality and common communicable diseases cannot be ignored. In hospitalized patients with community-acquired pneumonia, the CCI is a better predictor of in-hospital mortality than pneumonia-specific indices [36]. Additionally, allergic rhinitis has been associated with chronic systemic inflammatory activity and an increased risk of cardiovascular events and mortality, suggesting that it may reflect an underlying vulnerability relevant to mortality risk rather than a direct lethal condition [5]. Furthermore, prediction of mortality risk should consider the effect of COVID-19. To date, several risk factors have been identified as having a potential impact on the severity and mortality of COVID-19, including older age, male sex, and underlying comorbidities [40]. In particular, independent risk factors associated with mortality owing to COVID-19 include a history of chronic obstructive pulmonary disease, asthma, hypercholesterolemia, and type 2 diabetes [40,41]. A study in South Korea researched the effect of comorbidities on the infection rate and severity of COVID-19, in which CCI was used as a factor in propensity score matching [42].

Dyslipidemia is the most common cardiometabolic risk factor, followed by hypertension [38]. These results suggested that dyslipidemia and hypertension may be associated with mortality risk. However, these are not included neither in the CCI, nor in other comorbidity indices. Additionally, individuals with greater comorbidity had lower LDL-C levels than individuals with no comorbidities in this study (Table 3). The supplemental analyses showed that individuals diagnosed with dyslipidemia who were prescribed medication for dyslipidemia had lower LDL-C levels than those who were not prescribed medication for dyslipidemia. This suggested that statin therapy is highly effective for lowering LDL-C levels (Table S21). In contrast, individuals diagnosed with hypertension had higher SBP and DBP levels than those who were not diagnosed with hypertension, regardless of whether or not they were receiving pharmacotherapy. Additionally, individuals diagnosed with diabetes who were prescribed antidiabetic medication had higher HbA_1c_ levels than those who were not prescribed antidiabetic medication (Table S21). These results suggest that diet and exercise therapy are essential for improving hypertension and diabetes control, in addition to pharmacotherapy. To more precisely predict mortality rates, several complex factors such as major biochemical parameters and medication use should be considered.

The NDB dataset obtained for this study did not include Kampo medicines, so we were unable to directly assess their contribution to the overall medication burden. However, according to the publicly available 5th NDB Open Data Japan in 2018 [43], the proportion of Kampo medicines prescribed (calculated as the total quantity per prescription multiplied by the number of prescription days) accounted for 7.2% of all prescriptions across all age and sex groups. This suggests that the exclusion of Kampo medicines is unlikely to have substantially affected the results of the present study, although their use is relatively more common among older adults.

In this study, we determined CCI using both diagnosed diseases and prescribed medications from the Japanese NDB. Most previous studies have developed algorithms to determine CCI based only on disease diagnosis codes, using administrative claims databases [9,10,22]. However, the original CCI includes criteria that are not distinguishable using the ICD-10, such as pharmacotherapy. The CCI is a comorbidity index that is the most widely used for estimating mortality rate in patients with multiple comorbidities, and it was originally intended for use in longitudinal studies. Several studies have assessed the validity of the CCI as a predictor of mortality in certain individuals, such as those with acute myocardial infarction [44], coronary artery disease [45], ischemic stroke [46], hemodialysis and peritoneal dialysis [47], postoperative esophageal cancer [48], acute coronary syndrome [49], and frailty in elderly surgical patients [50]. Additionally, the CCI has been used in outcome studies to predict patient prognosis, including readmission in heart failure [4] and postoperative complications after gynecological cancer surgery [3,51,52]. However, a previous study using in-hospital discharge data suggested that five CCI comorbidities (myocardial infarction, peripheral vascular disease, cerebrovascular disease, peptic ulcer disease, and diabetes without chronic complications) were not associated with in-hospital mortality [53]. Understandably, patients survive longer today than they did when the original CCI was developed, owing to advances and improvements in treatments and medical technologies. In fact, various modified CCIs have been developed, which are reweighted indices of comorbidities [2,53]. In the near future, efforts are underway to develop more comprehensive and validated predictors for actual mortality using the most recent Japanese NDB. In the efforts, the disclosed predictors besides the CCI comorbidities should be considered, such as the COVID-19 pandemic, pharmacotherapy including Kampo medicines, and biochemical parameters.

### Limitations

This study has several limitations. First, some medications, including Kampo (herbal) medicines, as well as several CCI components were unavailable in the NDB, such as hospitalization details, New York Heart Association functional class, dialysis, and post-kidney transplant status, which may have led to incomplete assessments of comorbidity severity beyond diagnostic codes (Table S2). Second, some disease labels in the NDB were ambiguous, such as “liver dysfunction” and “diabetes mellitus,” and specialist confirmation was not feasible. This may have led to misclassification in claims-based diagnoses, thereby precluding sensitivity analyses based on validated diagnoses. However, to partially address this issue, we conducted sensitivity analyses by examining the internal consistency of diagnosed chronic metabolic diseases (hypertension, diabetes, and dyslipidemia) between 2016 and 2018 (Table S23). All three diseases showed high sensitivity, specificity, PPV, and NPV (≥0.8), and the kappa coefficients were all above 0.7, indicating acceptable diagnostic stability within the NDB. Third, mortality and other longitudinal outcome data were not available, which prevented direct validation of the CCI against actual outcomes. Consequently, we were also unable to assess the predictive performance of the CCI for mortality or to estimate the specific weights of individual conditions in this population. These issues should be addressed in further studies using linked outcome data. In addition, although this algorithm was based on validated definitions from Charlson, Quan, and Ono [1,6,17], we could not conduct an external validation of the adapted CCI algorithm against independent datasets. Fourth, diagnoses and prescribed medications were identified from a single claim, which may have overestimated prevalence. However, previous validation studies in Japan indicated that definitions using both diagnostic codes and prescription information showed high PPVs, even when derived from single-claim diagnoses [54,55,56]. Further sensitivity analyses using stricter definitions (e.g., ≥2 claims) or linkage with electronic medical record datasets would strengthen future research. Nevertheless, this coding algorithm for defining the CCI was more detailed than conventional methods based solely on ICD-10 codes, and previous studies have demonstrated its good predictive performance (c-statistic ≥ 0.70) for mortality [57]. Fifth, information on income level and hospital type was not available in the dataset, and residual confounding by such factors cannot be excluded. Additionally, surveillance bias, namely individuals with higher CCI are likely to have more frequent healthcare contact leading to increased diagnostic and prescribing opportunity, may have influenced the observed disease prevalence and medication use. Moreover, because the study population consisted of individuals who underwent specific health checkups and did not include welfare recipients, selection bias related to socioeconomic or occupational factors cannot be excluded. Addressing these limitations in future research will strengthen the reliability of the CCI in Japan and enhance its application in epidemiological studies, clinical practice, and health policy development. Sixth, individual-level ages were not available in the NDB, and only 5-year categories were provided. We therefore transformed these into substitute ages for analysis. Accordingly, age-adjusted CCI was calculated based on these categories, which may not fully reflect individual age differences. Finally, because this study is an exploratory survey of thousands of diseases and medications, formal corrections for multiple testing were not applied. These results should be interpreted cautiously and require confirmation in future hypothesis-driven studies that implement appropriate multiplicity control.

## 5. Conclusions

This study identified comorbidities and concomitant medications that are over-looked in conventional indices for predicting mortality, particularly allergic rhinitis and common communicable conditions such as acute bronchitis and upper respiratory infections. These results highlight potential opportunities to refine comorbidity indices by incorporating additional conditions that may influence prognoses. Furthermore, this analysis of a real-world Japanese administrative database demonstrated age- and CCI-specific patterns of comorbidities and pharmacotherapy, suggesting that the CCI may reflect aging processes while also supporting practitioners in identifying young individuals with multiple comorbidities who can be considered high risk. These findings are valuable for epidemiological studies using real-world databases and may inform health policy by providing novel insights into comorbidity, pharmacotherapy, clinical parameters, and underlying mechanisms concerning mortality. Moreover, these findings warrant further investigation to improve risk stratification and guide the development of more comprehensive comorbidity scoring systems in Japan.

## Figures and Tables

**Table 1:** Prevalence of 30 most-diagnosed diseases in all included individuals.

Order	Names of Diagnoses	Corresponding ICD-10 Code	N	%
1	Allergic rhinitis	J304	3,065,920	30.1
2	Hypertension	I10	2,273,580	22.3
3	Acute bronchitis	J209	1,720,087	16.9
4	Allergic conjunctivitis	H101	1,663,753	16.3
5	Astigmatism	H522	1,660,681	16.3
6	Acute upper respiratory infection	J069	1,379,966	13.6
7	Chronic gastritis	K295	1,308,946	12.9
8	Hyperlipidemia	E785	1,105,219	10.9
9	Asthmatic bronchitis	J459	1,022,058	10.0
10	Low back pain	M5456	1,013,491	10.0
11	Pure hypercholesterolemia	E780	994,931	9.77
12	Reflux esophagitis	K210	962,915	9.46
13	Gastritis	K297	959,187	9.42
14	Unspecified diabetes mellitus	E14	932,525	9.16
15	Acute laryngopharyngitis	J060	906,869	8.91
16	Sleep disorders	G470	868,176	8.53
17	Eczema	L309	790,078	7.76
18	Constipation	K590	775,587	7.62
19	Acute pharyngitis	J029	672,645	6.61
20	Dyslipidemia	E785	670,287	6.58
21	Gastric ulcer	K259	662,972	6.51
22	Pharyngitis	J029	613,103	6.02
23	Hyperuricemia	E790	610,144	5.99
24	Acute sinusitis	J019	597,165	5.86
25	Dry eye syndrome	H041	585,108	5.75
26	Cataract	H269	579,856	5.69
27	Gonarthrosis	M171	558,320	5.48
28	Disorder of peripheral nervous system	G629	512,869	5.04
29	Hypermetropic astigmatism	H522	509,477	5.00
30	Acute gastritis	K291	480,256	4.72

ICD-10: International Classification of Diseases, 10th Revision. The 30 most-diagnosed diseases among all included individuals.

**Table 2:** Prevalence of 30 most-prescribed medications in all included individuals.

Order	Therapeutic Category	Drug Name	N	%	Number of Generic Drugs
1	Antipyretics and analgesics, anti-inflammatory agents	Loxoprofen Sodium Hydrate 60 mg generic	1,625,762	16.0	18
2	Peptic ulcer agents	Rebamipide 100 mg generic	1,565,015	15.4	28
3	Expectorants	L-Carbocisteine 500 mg generic	1,242,743	12.2	5
4	Hemostatics	Tranexamic Acid 250 mg generic	832,427	8.17	3
5	Antipyretics and analgesics, anti-inflammatory agents	Acetaminophen 200 mg original	826,003	8.11	—
6	Acting mainly on Gram-positive bacteria and mycoplasma	Clarithromycin 200 mg generic	629,535	6.18	14
7	Other allergic agents	Fexofenadine Hydrochloride 60 mg generic	592,773	5.82	31
8	Antitussives	Dextromethorphan Hydrobromide Hydrate 15 mg generic	577,288	5.67	2
9	Common cold drugs	Salicylamide/Acetaminophen/Anhydrous Caffeine/Promethazine Methylenedisalicylate original	564,114	5.54	3
10	Other allergic agents	Montelukast Sodium 10 mg generic	543,802	5.34	39
11	Acting mainly on Gram-positive and Gram-negative bacteria	Cefcapene Pivoxil Hydrochloride Hydrate 100 mg generic	521,540	5.12	5
12	Expectorants	L-Carbocisteine 250 mg generic	488,884	4.80	5
13	Ophthalmic agents	Olopatadine Hydrochloride Solution 0.1% original	453,098	4.45	—
14	Antipyretics and analgesics, anti-inflammatory agents	Loxoprofen Sodium Hydrate 60 mg original	438,888	4.31	—
15	Other allergic agents	Bilastine 20 mg original	419,665	4.12	—
16	Antitussives	Dihydrocodeine Phosphate/dL-Methylephedrine Hydrochloride/ Chlorpheniramine Maleate original	393,262	3.86	—
17	Other allergic agents	Levocetirizine Hydrochloride 5 mg original	383,144	3.76	—
18	Other digestive organ agents	Dequalinium Chloride Troches 0.25 mg generic	362,891	3.56	—
19	Antitussives and expectorants	Tipepidine Hibenzate 20 mg original	345,287	3.39	—
20	Antipyretics and analgesics, anti-inflammatory agents	Acetaminophen 300 mg original	343,955	3.38	—
21	Antipyretics and analgesics, anti-inflammatory agents	Celecoxib 100 mg original	339,625	3.34	—
22	Hyperlipidemia agents	Rosuvastatin Calcium 2.5 mg generic	323,649	3.18	40
23	Antitussives	Dextromethorphan Hydrobromide Hydrate 15 mg original	318,049	3.12	—
24	Otic and nasal agents	Fluticasone Furoate 27.5 µg metered Nasal Spray original	310,090	3.04	—
25	Antipyretics and analgesics, anti-inflammatory agents	Acetaminophen 500 mg original	304,612	2.99	—
26	Other allergic agents	Bepotastine Besilate 10 mg generic	303,605	2.98	13
27	Expectorants	L-Carbocisteine 500 mg original	301,392	2.96	—
28	Anticoagulants	Heparinoid cream/lotion/spray/gel 0.3% generic	277,929	2.73	26
29	Antidiarrheals, intestinal regulators	Clostridium butyricum original	272,769	2.68	—
30	Peptic ulcer agents	Esomeprazole Magnesium Hydrate 20 mg original	252,680	2.48	—

The 30 most-prescribed medications are described according to their therapeutic category, nonproprietary name, whether they are original or generic products, and their dosage. Therapeutic category is decided according to the package insert or the Prescription Medications in Pharmaceuticals and Medical Devices Agency’s search system [20].

**Table 3:** Clinical characteristics of individuals according to CCI category.

	All	CCI = 0	CCI ≥ 4
N (%)	9,182,226	5,775,406 (62.9)	314,252 (3.42)
Men (n, %)	5,012,643 (54.6)	3,220,387 (55.8)	186,839 (59.5)
Women (n, %)	4,169,583 (45.4)	2,555,019 (44.2)	127,413 (40.5)
S-age (years old)	53.3 ± 10.0	51.6 ± 9.4	61.3 ± 8.7
BMI (kg/m^2^)	23.3 ± 3.7	23.0 ± 3.6	24.3 ± 4.3
SBP (mmHg)	123.5 ± 17.4	122.6 ± 17.5	127.8 ± 16.9
DBP (mmHg)	75.9 ± 11.9	75.8 ± 12.1	76.0 ± 11.2
TG (IQ)(mg/dL)	91 (64–135)	87 (61–130)	104 (73–152)
HDL-C (mg/dL)	64.4 ± 17.3	65.0 ± 17.3	60.6 ± 17.3
LDL-C (mg/dL)	124.8 ± 31.3	126.4 ± 31.3	114.3 ± 31.2
AST (U/L)	23.5 ± 10.6	22.8 ± 9.2	26.1 ± 13.9
ALT (U/L)	23.5 ± 16.8	22.5 ± 15.3	25.6 ± 19.1
γ-GTP (U/L)	39.2 ± 46.5	37.0 ± 42.2	45.3 ± 57.1
HbA_1c_ (%)	5.6 ± 0.6	5.5 ± 0.5	6.1 ± 1.0
CCI	0.68 ± 1.2	0.0	4.97 ± 1.35
Current smokers, n (%)	2,039,421 (22.2)	1,417,283 (24.5)	47,105 (15.0)
Regular exercise, n (%) ^*1^	2,418,722 (26.3)	2,094,536 (36.3)	97,079 (30.9)
Regular drinking, n (%) ^*2^	5,000,474 (54.5)	3,245,055 (56.2)	144,759 (46.1)
Diagnosed disease concerning CCI, n (%)			
Myocardial infarction	58,223 (0.63)	— ^*3^	24,653 (7.84)
Congestive heart failure	306,096 (3.33)	— ^*3^	96,306 (30.7)
Peripheral vascular disease	365,887 (3.98)	— ^*3^	90,018 (28.7)
Dementia	17,146 (0.19)	— ^*3^	4789 (1.52)
Cerebrovascular disease	432,804 (4.71)	— ^*3^	102,466 (32.6)
Chronic pulmonary disease	1,407,149 (15.3)	— ^*3^	143,261 (45.6)
Rheumatic disease	133,504 (1.45)	— ^*3^	29,384 (9.35)
Peptic ulcer disease	729,083 (7.94)	— ^*3^	141,332 (45.0)
Hemiplegia/paraplegia	15,775 (0.17)	— ^*3^	8865 (2.82)
Renal disease	81,098 (0.88)	— ^*3^	44,215 (14.1)
AIDS	793 (0.01)	— ^*3^	793 (0.25)
Mild liver disease	859,478 (9.36)	— ^*3^	146,447 (46.6)
Moderate or severe liver disease	7227 (0.08)	— ^*3^	5457 (1.74)
Diabetes without chronic complication	325,131 (3.54)	— ^*3^	53,467 (17.0)
Diabetes with chronic complication	222,362 (2.42)	— ^*3^	86,165 (27.4)
Tumor/lymphoma/leukemia	368,337 (4.01)	— ^*3^	111,033 (35.3)
Metastatic tumor	34,480 (0.38)	— ^*3^	34,480 (11.1)
Other diagnosed disease, n (%)			
Hypertension	2,160,064 (23.5)	738,737 (12.8)	209,935 (66.8)
Dyslipidemia	2,136,030 (23.3)	658,745 (11.4)	209,149 (66.6)
Hyperuricemia or Gout	672,985 (7.33)	216,817 (3.75)	76,534 (24.4)

Data are presented as mean ± standard deviation, median (interquartile range; IQ) for TG, or n (%). Statistical significance of difference between CCI = 0 and CCI ≥ 4 was compared using analysis of co-variance adjusting for S-age. Analysis of co-variance for categorical variables was conducted after conversion to continuous variables. Significant differences were observed in all continuous and categorical variables between CCI categories (all *p* < 0.0001). Note: ^*1^—regular exercise defined as ≥30 min at least twice a week; ^*2^—regular drinking defined as drinking every day or drinking occasionally; ^*3^—no individuals in this category. ALT, alanine aminotransferase; AST, aspartate aminotransferase; BMI, body mass index; CCI, Charlson Comorbidity Index; DBP, diastolic blood pressure; GTP, γ-glutamyl transferase; HbA_1c_, glycated hemoglobin; HDL-C, high-density lipoprotein cholesterol; LDL-C, low-density lipoprotein cholesterol; S-age, substituted age; SBP, systolic blood pressure; TG, triglycerides.

**Table 4:** Prevalence of diagnosed diseases in included individuals with CCI = 0.

Order	Age 40–44			Age 70–74			
	Names of Diagnoses	N	%	Names of Diagnoses	N	%	*p* vs. Age 40–44
1	Allergic rhinitis	272,164	23.4	Hypertension	150,073	32.7	<0.0001
2	Acute bronchitis	164,332	14.2	Allergic rhinitis	99,031	21.6	<0.0001
3	Astigmatism	162,935	14.0	Cataract	72,065	15.7	<0.0001
4	Allergic conjunctivitis	146,848	12.6	Allergic conjunctivitis	68,161	14.9	<0.0001
5	Acute upper respiratory infection	140,756	12.1	Astigmatism	64,738	14.1	0.03
6	Acute laryngopharyngitis	106,299	9.15	Hypermetropic astigmatism	60,468	13.2	<0.0001
7	Acute sinusitis	77,377	6.66	Chronic gastritis	59,937	13.1	<0.0001
8	Acute pharyngitis	73,295	6.31	Hyperlipidemia	59,789	13.0	<0.0001
9	Gastritis	66,416	5.72	Hypercholesterolemia	59,655	13.0	<0.0001
10	Chronic gastritis	61,911	5.33	Osteoporosis	53,528	11.7	<0.0001
11	Influenza A	60,075	5.17	Acute bronchitis	52,818	11.5	<0.0001
12	Eczema	59,120	5.09	Sleep disorders	50,311	11.0	<0.0001
13	Acute gastritis	51,454	4.43	Gonarthrosis	49,365	10.8	<0.0001
14	Pharyngitis	50,970	4.39	Low back pain	48,351	10.5	<0.0001
15	Low back pain	48,970	4.22	Acute upper respiratory infection	43,928	9.58	<0.0001
16	Reflux esophagitis	38,610	3.33	Gastritis	40,727	8.88	<0.0001
17	Sleep disorders	36,507	3.14	Reflux esophagitis	40,289	8.79	<0.0001
18	Atopic dermatitis	36,239	3.12	Constipation	38,816	8.47	<0.0001
19	Asteatosis	33,717	2.90	Dry eye syndrome	38,222	8.34	<0.0001
20	Asteatosis eczema	33,384	2.88	Eczema	36,761	8.02	<0.0001
21	Dry eye syndrome	33,104	2.85	Dyslipidemia	33,061	7.21	<0.0001
22	Hypertension	33,087	2.85	Unspecified diabetes mellitus	32,706	7.13	<0.0001
23	Conjunctivitis	32,106	2.77	Conjunctivitis	29,533	6.44	<0.0001
24	Common cold	30,733	2.65	Presbyopia	27,741	6.05	<0.0001
25	Leiomyoma of uterus	30,723	2.65	Glaucoma	25,718	5.61	<0.0001
26	Iron deficiency anemia	29,392	2.53	Lumbar spondylosis deformans	24,741	5.40	<0.0001
27	Constipation	28,894	2.49	Acute laryngopharyngitis	24,457	5.33	<0.0001
28	Influenza, virus not identified	28,531	2.46	Lumbar spinal stenosis	23,778	5.19	<0.0001
29	Diarrhea	28,295	2.44	Hyperplasia of prostate	23,515	5.13	<0.0001
30	Chronic sinusitis	27,659	2.38	Disorder of peripheral nervous system	23,267	5.07	<0.0001

The 30 most-diagnosed diseases and conditions in all included individuals are described. *p* values between age categories were calculated by using Student’s *t*-test. Student’s *t*-test was conducted after conversion to continuous variables. ICD-10: International Classification of Diseases, 10th Revision.

**Table 5:** Prevalence of diagnosed diseases in the individuals with CCI ≥ 4 (age-adjusted CCI ≥ 5 and CCI ≥ 8 in aged 40–44 and 70–74).

Order	Age 40–44			Age 70–74			
	Names of Diagnoses	N	%	Names of Diagnoses	N	%	*p* vs. Age 40–44
1	Allergic rhinitis	5471	52.5	Hypertension	75,155	72.2	<0.0001
2	Hypertension	4155	39.9	Unspecified diabetes mellitus	50,906	48.9	<0.0001
3	Asthmatic bronchitis	3598	34.5	Allergic rhinitis	45,100	43.3	<0.0001
4	Unspecified diabetes mellitus	3564	34.2	Hyperlipidemia	42,306	40.6	<0.0001
5	Gastric ulcer	3557	34.2	Gastric ulcer	41,390	39.8	<0.0001
6	Acute bronchitis	3271	31.4	Chronic gastritis	38,749	37.2	<0.0001
7	Astigmatism	3064	29.4	Constipation	38,507	37.0	<0.0001
8	Chronic gastritis	2917	28.0	Hypercholesterolemia	35,818	34.4	<0.0001
9	Low back pain	2864	27.5	Low back pain	32,468	31.2	<0.0001
10	Constipation	2718	26.1	Reflux esophagitis	32,060	30.8	<0.0001
11	Acute upper respiratory infection	2682	25.8	Sleep disorders	31,490	30.2	<0.0001
12	Hyperlipidemia	2631	25.3	Cataract	27,519	26.4	<0.0001
13	Allergic conjunctivitis	2592	24.9	Asthmatic bronchitis	26,650	25.6	<0.0001
14	Sleep disorders	2416	23.2	Astigmatism	26,590	25.5	<0.0001
15	Reflux esophagitis	2285	21.9	Type 2 diabetes mellitus	25,688	24.7	<0.0001
16	Hyperuricemia	2247	21.6	Allergic conjunctivitis	25,030	24.0	n.s
17	Liver dysfunction	2142	20.6	Angina pectoris	24,427	23.5	<0.0001
18	Iron deficiency anemia	2111	20.3	Acute bronchitis	24,399	23.4	<0.0001
19	Hypercholesterolemia	2080	20.0	Osteoporosis	24,310	23.3	<0.0001
20	Type 2 diabetes mellitus	2027	19.5	Intractable reflux esophagitis with maintenance therapy	23,889	22.9	<0.0001
21	Fatty liver	1944	18.7	Hypermetropic astigmatism	23,229	22.3	<0.0001
22	Gastritis	1767	17.0	Dyslipidemia	21,834	21.0	<0.0001
23	Eczema	1766	17.0	Hyperuricemia	21,227	20.4	<0.0001
24	Acute laryngopharyngitis	1733	16.6	Hyperplasia of prostate	19,876	19.1	<0.0001
25	Dyslipidemia	1679	16.1	Gonarthrosis	19,659	18.9	<0.0001
26	Pharyngitis	1564	15.0	Gastritis	19,308	18.5	<0.0001
27	Intractable reflux esophagitis with maintenance therapy	1546	14.8	Fatty liver	19,300	18.5	n.s
28	Bronchitis	1524	14.6	Eczema	18,664	17.9	<0.0001
29	Acute pharyngitis	1293	12.4	Disorder of peripheral nervous system	18,571	17.8	<0.0001
30	Asteatosis	1226	11.8	Acute upper respiratory infection	18,374	17.6	<0.0001

The 30 most-diagnosed diseases and conditions in the included individuals with CCI ≥ 4 (age-adjusted CCI ≥ 5 and CCI ≥ 8 in individuals aged 40–44 and 70–74) are described. *p* values between age categories were calculated by using Student’s *t*-test. Student’s *t*-test was conducted after conversion to continuous variables. ICD-10: International Classification of Diseases, 10th Revision.

**Table 6:** The prevalence of diagnosed diseases between CCI category (0 or ≥4) and age group (40–44 or 70–74 years).

	CCI = 0		CCI ≥ 4	
Diagnosed Diseases	Age 40–44 n = 1,161,138	Age 70–74 n = 458,479	Age 40–44 n = 10,417	Age 70–74 n = 104,115
Type 2 diabetes mellitus	2389 (0.21)	7182 (1.57)	2027 (19.5)	25,688 (24.7)
Hypertension	33,087 (2.85)	150,073 (32.7)	4156 (39.9)	75,156 (72.2)
Hyperlipidemia	17,031 (1.47)	59,789 (13.0)	2631 (25.3)	42,307 (40.6)
Iron deficiency anemia	29,392 (2.53)	5678 (1.24)	2113 (20.3)	14,385 (13.8)
Hypothyroidism	4937 (0.43)	3992 (0.87)	769 (7.38)	5779 (5.55)
Intractable reflux esophagitis	6193 (0.53)	15,361 (3.35)	1547 (14.9)	23,890 (23.0)
Gastritis	66,416 (5.72)	40,727 (8.88)	1767 (17.0)	19,308 (18.5)
Constipation	28,894 (2.49)	38,816 (8.47)	2718 (26.1)	38,508 (37.0)
Diarrhea	28,295 (2.44)	7809 (1.70)	1151 (11.1)	6928 (6.65)
Depressive episodes	22,103 (1.90)	7318 (1.60)	870 (8.35)	5660 (5.44)
Sleep disorders	36,507 (3.14)	50,311 (11.1)	2416 (23.2)	31,490 (30.3)
Anxiety neurosis	11,596 (1.00)	11,815 (2.58)	684 (6.57)	8418 (8.09)

**Table 7:** Prevalence of prescribed medications in the individuals with CCI = 0.

Order	Age 40–44			Age 70–74			
	Drug Name	N	%	Drug Name	N	%	*p* vs. Age 40–44
1	Loxoprofen Sodium Hydrate 60 mg generic	167,937	14.5	Rebamipide 100 mg generic	57,911	12.6	<0.0001
2	Rebamipide 100 mg generic	136,023	11.7	Loxoprofen Sodium Hydrate 60 mg generic	50,465	11.0	<0.0001
3	L-Carbocisteine 500 mg generic	129,329	11.1	L-Carbocisteine 500 mg generic	29,856	6.51	<0.0001
4	Tranexamic Acid 250 mg generic	94,081	8.10	Loxoprofen Sodium Hydrate Tape/Patch 100 mg generic	29,591	6.45	<0.0001
5	Acetaminophen 200 mg original	93,904	8.09	Acetaminophen 200 mg original	22,217	4.85	<0.0001
6	Clarithromycin 200 mg generic	58,155	5.01	Celecoxib 100 mg original	21,269	4.64	<0.0001
7	Fexofenadine Hydrochloride 60 mg generic	57,177	4.92	Tranexamic Acid 250 mg generic	20,558	4.48	<0.0001
8	Dextromethorphan Hydrobromide Hydrate generic	53,938	4.65	Salicylamide/Acetaminophen/Anhydrous Caffeine/Promethazine Methylenedisalicylate original	18,862	4.11	n.s
9	Cefcapene Pivoxil Hydrochloride Hydrate 100 mg generic	53,901	4.64	Cefcapene Pivoxil Hydrochloride Hydrate 100 mg generic	18,316	3.99	<0.0001
10	Salicylamide/Acetaminophen/Anhydrous Caffeine/Promethazine Methylenedisalicylate original	48,128	4.14	Rosuvastatin Calcium 2.5 mg generic	18,241	3.98	<0.0001
11	L-Carbocisteine 250 mg generic	48,103	4.14	Fexofenadine Hydrochloride 60 mg generic	17,850	3.89	<0.0001
12	Bilastine 20 mg original	45,106	3.88	Olopatadine Hydrochloride Solution 0.1% original	17,764	3.87	<0.0001
13	Cefditoren Pivoxil 100 mg generic	44,726	3.85	Eldecalcitol 0.75 µg original	16,437	3.59	<0.0001
14	Olopatadine Hydrochloride Solution 0.1% original	40,048	3.45	Dextromethorphan Hydrobromide Hydrate 15 mg generic	15,710	3.43	<0.0001
15	Levocetirizine Hydrochloride 5 mg original	39,337	3.39	L-Carbocisteine 250 mg generic	14,573	3.18	<0.0001
16	Loxoprofen Sodium Hydrate 60 mg original	38,658	3.33	Loxoprofen Sodium Hydrate 60 mg original	14,506	3.16	<0.0001
17	Acetaminophen 300 mg original	36,784	3.17	Diquafosol Sodium Solution 3% original	12,164	2.65	<0.0001
18	Acetaminophen 500 mg original	35,377	3.05	Ketoprofen Tapes 40 mg original	11,865	2.59	<0.0001
19	Fluticasone Furoate 27.5 µg metered Nasal Spray original	34,322	2.96	Mecobalamin 500 µg original	11,560	2.52	<0.0001
20	Tipepidine Hibenzate 20 mg original	33,988	2.93	Heparinoid cream/lotion/spray/gel 0.3% generic	11,426	2.49	<0.0001
21	Dequalinium Chloride Troches 0.25 mg generic	33,605	2.89	Bilastine 20 mg original	11,089	2.42	<0.0001
22	Montelukast Sodium 10 mg generic	33,553	2.89	Dequalinium Chloride 0.25 mg generic	10,812	2.36	<0.0001
23	Bepotastine Besilate 10 mg generic	32,419	2.79	Betamethasone Valerate/Gentamicin Sulfate Ointment 0.12% original	10,607	2.31	<0.0001
24	Dihydrocodeine Phosphate/dL-Methylephedrine Hydrochloride/ Chlorpheniramine Maleate original	32,400	2.79	Levocetirizine Hydrochloride 5 mg original	10,479	2.29	<0.0001
25	Baloxavir Marboxil 20 mg original	32,280	2.78	Loxoprofen Sodium Hydrate Tapes 100 mg original	10,412	2.27	<0.0001
26	Clostridium butyricum original	28,308	2.44	Dextromethorphan Hydrobromide Hydrate 15 mg original	9813	2.14	<0.05
27	Tranexamic Acid 500 mg generic	28,023	2.41	Rebamipide 100 mg original	9491	2.07	<0.0001
28	L-Carbocisteine 500 mg original	26,753	2.30	Acetaminophen 300 mg original	9362	2.04	<0.0001
29	Garenoxacin Mesilate Hydrate 200 mg original	26,159	2.25	Tipepidine Hibenzate 20 mg original	9247	2.02	<0.0001
30	Antibiotics-Resistant Lactic Acid Bacteriae original	25,988	2.24	Heparinoid Ointment 0.3% original	8859	1.93	<0.0001

The 30 most-prescribed medications are described according to their therapeutic category, nonproprietary names, whether they are original or generic products, and their dosage. *p* values between age categories were calculated by using the χ^2^ test.

**Table 8:** Prevalence of prescribed medications in the individuals with CCI ≥ 4 (age-adjusted CCI ≥ 5 and CCI ≥ 8 in individuals aged 40–44 and 70–74).

Order	Age 40–44			Age 70–74			
	Drug Name	N	%	Drug Name	N	%	*p* vs. Age 40–44
1	Loxoprofen Sodium Hydrate 60 mg generic	3059	29.4	Rebamipide 100 mg generic	22,923	22.0	<0.0001
2	Rebamipide 100 mg generic	2784	26.7	Loxoprofen Sodium Hydrate 60 mg generic	19,471	18.7	<0.0001
3	L-Carbocisteine 500 mg generic	2549	24.5	L-Carbocisteine 500 mg generic	15,925	15.3	<0.0001
4	Acetaminophen 200 mg original	1785	17.1	Aspirin 100 mg original	13,401	12.9	<0.0001
5	Tranexamic Acid 250 mg generic	1679	16.1	Acetaminophen 200 mg original	10,970	10.5	<0.0001
6	Montelukast Sodium 10 mg generic	1450	13.9	Rosuvastatin Calcium 2.5 mg generic	9777	9.39	<0.0001
7	Clarithromycin generic	1310	12.6	Esomeprazole Magnesium Hydrate 20 mg original	9270	8.90	<0.0001
8	Dextromethorphan Hydrobromide Hydrate 15 mg generic	1192	11.4	Salicylamide/Acetaminophen/Anhydrous Caffeine/Promethazine Methylenedisalicylate original	9007	8.65	<0.0001
9	Salicylamide/Acetaminophen/Anhydrous Caffeine/Promethazine Methylenedisalicylate original	1173	11.26	Tranexamic Acid 250 mg generic	8505	8.17	<0.0001
10	Cefcapene Pivoxil Hydrochloride Hydrate 100 mg generic	1002	9.62	Celecoxib 100 mg original	8319	7.99	<0.0001
11	L-Carbocisteine 250 mg generic	943	9.05	Dextromethorphan Hydrobromide Hydrate 15 mg generic	7964	7.65	<0.0001
12	Loxoprofen Sodium Hydrate 60 mg original	923	8.86	Cefcapene Pivoxil Hydrochloride Hydrate 100 mg generic	7599	7.30	<0.0001
13	Dihydrocodeine Phosphate/dL-Methylephedrine Hydrochloride/ Chlorpheniramine Maleate original	909	8.73	L-Carbocisteine 250 mg generic	7390	7.10	<0.0001
14	Acetaminophen 500 mg original	888	8.53	Montelukast Sodium 10 mg generic	7208	6.92	<0.0001
15	Bilastine 20 mg original	819	7.86	Mecobalamin 500 µg original	6927	6.65	<0.0001
16	Acetaminophen 300 mg original	798	7.66	Magnesium Oxide 330 mg generic	6579	6.32	<0.0001
17	Dequalinium Chloride Troches 0.25 mg generic	775	7.44	Loxoprofen Sodium Hydrate 60 mg original	6391	6.14	<0.0001
18	Clostridium butyricum original	772	7.41	Olopatadine Hydrochloride Solution 0.1% original	6260	6.01	<0.05
19	Dextromethorphan Hydrobromide Hydrate 15 mg original	770	7.39	Ketoprofen Tapes 40 mg original	6241	5.99	<0.0001
20	Esomeprazole Magnesium Hydrate 20 mg original	770	7.39	Heparinoid cream/lotion/spray/gel 0.3% generic	6130	5.89	n.s
21	Levocetirizine Hydrochloride 5 mg original	769	7.38	Eldecalcitol 0.75 µg original	5546	5.33	<0.0001
22	L-Carbocisteine 500 mg original	753	7.23	Dextromethorphan Hydrobromide Hydrate 15 mg original	5530	5.31	<0.0001
23	Fluticasone Furoate 27.5 µg metered Nasal Spray original	748	7.18	Sitagliptin Phosphate Hydrate 50 mg original	5426	5.21	<0.0001
24	Olopatadine Hydrochloride Solution 0.1% original	704	6.76	Dequalinium Chloride Troches 0.25 mg generic	5393	5.18	<0.0001
25	Tipepidine Hibenzate 20 mg original	690	6.63	Vonoprazan Fumarate 10 mg original	5375	5.16	<0.0001
26	Heparinoid Cream/Lotion/Spray/Gel 0.3% generic	662	6.36	Febuxostat 10 mg original	5188	4.98	<0.001
27	Rosuvastatin Calcium 2.5 mg generic	635	6.10	Loxoprofen Sodium Hydrate Tapes 100 mg original	5094	4.89	<0.0001
28	Prednisolone 5 mg original	633	6.08	Acetaminophen 300 mg original	5086	4.89	<0.0001
29	Garenoxacin Mesilate Hydrate 200 mg original	628	6.03	L-Carbocisteine 500 mg original	4962	4.77	<0.0001
30	Febuxostat 10 mg original	597	5.73	Metformin Hydrochloride 250 mg original	4853	4.66	n.s

The 30 most-prescribed medications are described according to their therapeutic categories, nonproprietary names, whether they are original or generic products, and their dosage. *p* values between age categories were calculated by using the χ^2^ test.

## Data Availability

The datasets presented in this article are not readily available because access to the National Database is regulated by the MHLW of Japan. Requests to access the datasets should be directed to the corresponding author.

## Supplementary Materials

The following supporting information can be downloaded at: https://www.mdpi.com/article/10.3390/epidemiologia7020034/s1, Figure S1: Conceptual model of the study; Table S1: Percentile values of clinical parameters from the NDB in the seven prefectures of the Kanto region; Table S2: Definitions of Charlson Comorbidity Index; Table S3: Prevalence of diagnosed diseases in all included individuals; Table S4: Prevalence of prescribed medications in all included individuals; Table S5: Prevalence of diagnosed diseases in the CCI = 0, 40–44 age group; Table S6: Prevalence of diagnosed diseases in the CCI = 0, 70–74 age group; Table S7: Prevalence of diagnosed diseases in the CCI ≥ 4, 40–44 age group; Table S8: Prevalence of diagnosed diseases in the CCI ≥ 4, 70–74 age group; Table S9: Prevalence of prescribed medications in the CCI = 0, 40–44 age group; Table S10: Prevalence of prescribed medications in the CCI = 0, 70–74 age group; Table S11: Prevalence of prescribed medications in the CCI ≥ 4, 40–44 age group; Table S12: Prevalence of prescribed medications in the CCI ≥ 4, 70–74 age group; Table S13: Prevalence of diagnosed diseases among men aged 40–44 years; Table S14: Prevalence of diagnosed diseases among men aged 70–74 years; Table S15: Prevalence of diagnosed diseases among women aged 40–44 years; Table S16: Prevalence of diagnosed diseases among women aged 70–74 years; Table S17: Prevalence of prescribed medications among men aged 40–44 years; Table S18: Prevalence of prescribed medications among men aged 70–74 years; Table S19: Prevalence of prescribed medications among women aged 40–44 years; Table S20: Prevalence of prescribed medications among women aged 70–74 years; Table S21: Proportion of individuals diagnosed among those prescribed specific medications; Table S22: Associations of CCI ≥ 4 in age 40–44 group with diagnosed diseases compared with CCI = 0 in age 70–74 group; Table S23: Consistency of diagnosed diseases between 2016 and 2018 (sensitivity, specificity, and kappa coefficient).

## Abbreviations

ALT	alanine aminotransferase
AST	aspartate aminotransferase
BMI	body mass index
CCI	Charlson Comorbidity Index
DBP	diastolic blood pressure
DKA	diabetic ketoacidosis
GTP	γ-glutamyl transferase
HbA_1c_	glycated hemoglobin
HDL-C	high-density lipoprotein cholesterol
HHS	hyperosmolar hyperglycemic syndrome
ICD-10	International Classification of Diseases, 10th Revision
LDL-C	low-density lipoprotein cholesterol
MHLW	Japan’s Ministry of Health, Labour and Welfare
NDB	The Japan National Database of Health Insurance Claims and Specific Health Checkups
S-age	substituted age
SBP	systolic blood pressure
TG	triglycerides
