# Supplementary material for: Comorbidities and Concomitant Medications in Middle-Aged Japanese People According to the Charlson Comorbidity Index and Age: Results of the NDB-K7Ps-Study-3
Source: Epidemiologia (Basel). 2026 Mar 2;7(2):34. doi: 10.3390/epidemiologia7020034 (PMC13010749; doi:10.3390/epidemiologia7020034)
Supplement: Supplementary file 1 [file epidemiologia-07-00034-s001.zip › Table S1.pdf]

Table S1. Percentile values of clinical parameters from the NDB in the seven prefectures of the Kanto region

| | 0.1st | 99.9th | 0.05th | 99.5th | 0.01st | 99.99th |
|---|---|---|---|---|---|---|
| BMI ($kg/m^2$) | 14.8 | 42.0 | 14.3 | 44.3 | 13.1 | 50.1 |
| SBP (mmHg) | 80 | 196 | 79 | 204 | 74 | 222 |
| DBP (mmHg) | 44 | 122 | 41 | 127 | 37 | 136 |
| TG (mg/dL) | 24 | 984 | 23 | 1204 | 19 | 1681 |
| HDL-C (mg/dL) | 28 | 139 | 26 | 147 | 21 | 173 |
| LDL-C (mg/dL) | 39 | 250 | 34 | 264 | 25 | 308 |
| AST (U/L) | 10 | 139 | 9 | 175 | 7 | 302 |
| ALT (U/L) | 5 | 173 | 4 | 207 | 3 | 341 |
| γ-GTP (U/L) | 7 | 563 | 7 | 684 | 5 | 900 |

Data are presented as the 0.1st, 99.9th, 0.05th, 99.5th, 0.01st, and 99.99th percentiles for each clinical parameter. The data include individuals aged 40–74 years who resided in any of the seven prefectures of the Kanto region and underwent specific health checkups between April 2018 and March 2019.

ALT, alanine aminotransferase; AST, aspartate aminotransferase; BMI, body mass index; DBP, diastolic blood pressure; GTP, γ-glutamyl transferase; HDL-C, high-density lipoprotein cholesterol; LDL-C, low-density lipoprotein cholesterol; S-age, substituted age; SBP, systolic blood pressure; TG, triglycerides.
