# Supplementary material for: Comorbidities and Concomitant Medications in Middle-Aged Japanese People According to the Charlson Comorbidity Index and Age: Results of the NDB-K7Ps-Study-3
Source: Epidemiologia (Basel). 2026 Mar 2;7(2):34. doi: 10.3390/epidemiologia7020034 (PMC13010749; doi:10.3390/epidemiologia7020034)
Supplement: Supplementary file 1 [file epidemiologia-07-00034-s001.zip › Table S13-20.pdf]

Table S13. Prevalence of diagnosed diseases among men aged 40-44 years

Order	Names of Diagnoses	Corresponding ICD-10 Code	N	%
1	Allergic rhinitis	J304	263,187	25.9
2	Acute bronchitis	J209	172,166	17.0
3	Acute upper respiratory infection	J069	146,825	14.5
4	Astigmatism	H522	118,659	11.7
5	Allergic conjunctivitis	H101	112,624	11.1
6	Acute laryngopharyngitis	J060	95,491	9.41
7	Asthmatic bronchitis	J459	83,200	8.20
8	Acute pharyngitis	J029	73,039	7.20
9	Hypertension	I10	68,258	6.73
10	Gastritis	K297	67,828	6.68
11	Chronic gastritis	K295	67,482	6.65
12	Acute sinusitis	J019	65,556	6.46
13	Pharyngitis	J029	60,771	5.99
14	Influenza A	J101	56,790	5.60
15	Low back pain	M5456	51,648	5.09
16	Reflux esophagitis	K210	50,615	4.99
17	Acute gastritis	K291	49,576	4.88
18	Hyperuricemia	E790	46,222	4.55
19	Eczema	L309	44,965	4.43
20	Sleep disorders	G470	41,623	4.10
21	Hyperlipidemia	E785	41,166	4.06
22	Bronchitis	J40	39,940	3.94
23	Common cold	J00	33,965	3.35
24	Atopic dermatitis	L209	33,666	3.32
25	Hypercholesterolemia	E780	32,581	3.21
26	Dyslipidemia	E785	32,514	3.20
27	Unspecified diabetes mellitus	E14	32,486	3.20
28	Diarrhea	A099	32,434	3.20
29	Gastric ulcer	K259	30,448	3.00
30	Liver dysfunction	K769	28,470	2.81
31	Chronic sinusitis	J329	27,954	2.75
32	Influenza, virus not identified	J111	27,089	2.67
33	Tonsillitis	J039	26,485	2.61
34	Conjunctivitis	H109	26,065	2.57
35	Fatty liver	K760	25,604	2.52
36	Depressive episode	F329	25,148	2.48
37	Asteatosis	L853	24,440	2.41

38	Asteatosis eczema	L853	23,664	2.33
39	Acute tonsillitis	J039	23,621	2.33
40	Disorder of peripheral nervous system	G629	23,404	2.31
41	Laryngopharyngitis	J060	23,070	2.27
42	Local infection of skin	L089	22,470	2.21
43	Constipation	K590	21,363	2.10
44	Urticaria	L509	20,429	2.01
45	Dehydration	E86	19,186	1.89
46	Dry eye syndrome	H041	18,160	1.79
47	Tinea pedis	B353	17,836	1.76
48	Type 2 diabetes mellitus	E11	16,329	1.61
49	lumbar disc herniation	M512	15,825	1.56
50	Gout	M1009	15,775	1.55
51	Myopia	H521	15,445	1.52
52	Neurotic disorder	F489	14,652	1.44
53	lumbar disc disorder	M519	14,604	1.44
54	Seborrheic dermatitis	L219	14,037	1.38
55	Intractable reflux esophagitis with maintenance therapy	K210	13,927	1.37
56	Sleep apnea	G473	13,350	1.32
57	Acute laryngitis	J040	13,322	1.31
58	Pollinosis	J301	13,210	1.30
59	Impacted cerumen	H612	13,115	1.29
60	Enlarged optic nerve head excavation	H400	12,924	1.27
61	Dermatitis	L309	12,910	1.27
62	Presbyopia	H524	12,387	1.22
63	Acute dermatitis	L309	12,086	1.19
64	Sinusitis	J329	11,854	1.17
65	Anxiety neurosis	F411	11,751	1.16
66	Internal hemorrhoid	K649	11,729	1.16
67	Glaucoma	H409	11,607	1.14
68	Contact dermatitis	L259	11,298	1.11
69	Polyp of colon	K635	11,289	1.11
70	Atrophic gastritis	K294	11,186	1.10
71	High myopia	H521	11,160	1.10
72	Helicobacter pylori infection	A498	10,875	1.07
73	Asthenopia	H531	10,702	1.05
74	Acne vulgaris	L700	10,600	1.04
75	Migraine	G439	10,349	1.02
76	Keratoconjunctivitis sicca	H168	10,164	1.00
77	Irritable bowel syndrome	K589	9,896	0.98

78	Gonarthrosis	M171	9,844	0.97
79	Neuropathic pain	G98	9,761	0.96
80	Otitis externa	H609	9,609	0.95
81	Blepharitis	H010	9,393	0.93
82	Schizophrenia	F209	9,091	0.90
83	Myalgia	M7919	8,774	0.86
84	Cervical spondylosis	M4782	8,737	0.86
85	Acute conjunctivitis	H103	8,686	0.86
86	Chronic bronchitis	J42	8,585	0.85
87	Pruritus	L299	8,538	0.84
88	Depressive episode	F329	8,508	0.84
89	Stomatitis	K121	8,363	0.82
90	Hypermetropic astigmatism	H522	8,299	0.82
91	Asthmatic bronchitis	J459	7,828	0.77
92	Chronic dermatitis	L309	7,819	0.77
93	Calculus of ureter	N201	7,767	0.77
94	Acute rhinitis	J00	7,724	0.76
95	Otitis externa eczematoid	H605	7,674	0.76
96	Acute exacerbation of chronic sinusitis	J329	7,383	0.73
97	Angina pectoris	I209	7,354	0.72
98	Hordeolum	H000	7,166	0.71
99	Peripheral neuropathic pain	G64	7,073	0.70
100	Intractable reflux esophagitis	K210	7,044	0.69

The 100 most-diagnosed diseases (or those with >1% prevalence) among men aged 40-44 years are described.

among men aged 40-44 years

ICD-10, International Classification of Diseases, 10th Revision.

Table S14. Prevalence of diagnosed diseases among men aged 70-74 years

Order	Names of Diagnoses	Corresponding ICD-10 Code	N	%
1	Hypertension	I10	277,758	52.6
2	Allergic rhinitis	J304	153,773	29.1
3	Unspecified diabetes mellitus	E14	121,747	23.1
4	Hyperlipidemia	E785	113,167	21.4
5	Chronic gastritis	K295	106,344	20.2
6	Hyperplasia of prostate	N40	105,821	20.1
7	Hypercholesterolemia	E780	94,135	17.8
8	Astigmatism	H522	87,924	16.7
9	Reflux esophagitis	K210	86,979	16.5
10	Hyperuricemia	E790	86,390	16.4
11	Cataract	H269	86,107	16.3
12	Low back pain	M5456	84,220	16.0
13	Constipation	K590	81,795	15.5
14	Acute bronchitis	J209	81,127	15.4
15	Allergic conjunctivitis	H101	77,880	14.8
16	Sleep disorders	G470	68,199	12.9
17	Hypermetropic astigmatism	H522	67,391	12.8
18	Gastric ulcer	K259	65,691	12.4
19	Acute upper respiratory infection	J069	63,385	12.0
20	Dyslipidemia	E785	59,783	11.3
21	Gastritis	K297	58,653	11.1
22	Eczema	L309	53,704	10.2
23	Angina pectoris	I209	53,333	10.1
24	Type 2 diabetes mellitus	E11	53,260	10.1
25	Intractable reflux esophagitis with maintenance therapy	K210	52,535	10.0
26	Asthmatic bronchitis	J459	51,473	9.8
27	Polyp of colon	K635	45,383	8.6
28	Gonarthrosis	M171	45,284	8.6
29	Disorder of peripheral nervous system	G629	43,007	8.1
30	Lumbar spinal stenosis	M4806	39,214	7.4
31	Conjunctivitis	H109	36,512	6.9
32	Atrophic gastritis	K294	36,020	6.8
33	Glaucoma	H409	34,963	6.6
34	Fatty liver	K760	34,421	6.5
35	Dry eye syndrome	H041	34,343	6.5
36	Acute laryngopharyngitis	J060	33,943	6.4
37	Lumbar spondylosis deformans	M4786	32,574	6.2

38	Liver dysfunction	K769	32,202	6.1
39	Presbyopia	H524	32,032	6.1
40	Asteatotic eczema	L853	31,869	6.0
41	Overactive bladder	N328	29,602	5.6
42	Tinea pedis	B353	28,743	5.4
43	Pharyngitis	J029	28,723	5.4
44	Acute pharyngitis	J029	27,993	5.3
45	Gout	M1009	26,556	5.0
46	Asteatosis	L853	26,012	4.9
47	Intractable reflux esophagitis	K210	25,793	4.9
48	Cerebral infarction	I639	24,730	4.7
49	Malignant neoplasm of prostate	C61	24,357	4.6
50	Asthenopia	H531	23,983	4.5
51	Chronic heart failure	I509	23,190	4.4
52	Iron deficiency anemia	D509	23,096	4.4
53	Bronchitis	J40	22,898	4.3
54	Unspecified diabetes retinopathy	E143	22,217	4.2
55	Cardiac arrhythmia	I499	22,037	4.2
56	Heart failure	I509	21,556	4.1
57	Senile cataract	H259	21,443	4.1
58	Chronic bronchitis	J42	21,212	4.0
59	Acute sinusitis	J019	20,953	4.0
60	Helicobacter pylori infection	A498	20,796	3.9
61	Atrial fibrillation	I489	19,381	3.7
62	Arteriosclerosis obliterans	I709	19,100	3.6
63	Keratoconjunctivitis sicca	H168	18,837	3.6
64	Chronic sinusitis	J329	18,833	3.6
65	Enlarged optic nerve head excavation	H400	18,162	3.4
66	Neuropathic pain	G98	18,050	3.4
67	Common cold	J00	17,829	3.4
68	Mixed astigmatism	H522	17,515	3.3
69	Dehydration	E86	17,399	3.3
70	Acute gastritis	K291	17,387	3.3
71	Osteoporosis	M8199	17,285	3.3
72	Essential hypertension	I10	17,161	3.3
73	Dermatitis	L309	16,243	3.1
74	Myalgia	M7919	15,935	3.0
75	Seborrheic dermatitis	L219	15,856	3.0
76	Peripheral neuropathic pain	G64	15,598	3.0
77	Internal hemorrhoid	K649	15,369	2.9

78	Diarrhea	A099	15,215	2.9
79	Cervical spondylosis deformans	M4782	15,207	2.9
80	Impacted cerumen	H612	15,027	2.8
81	Carotid artery stenosis	I652	14,898	2.8
82	Anxiety neurosis	F411	14,801	2.8
83	Local infection of skin	L089	14,544	2.8
84	Anemia	D649	14,479	2.7
85	Polyp of stomach	K317	14,041	2.7
86	Cyst of kidney	N281	14,032	2.7
87	influenza A	J101	13,865	2.6
88	Calculus of gallbladder	K802	13,826	2.6
89	Urticaria	L509	13,580	2.6
90	Contact dermatitis	L259	13,553	2.6
91	Cervical spondylosis	M4782	13,400	2.5
92	Nonvalvular atrial fibrillation	I489	13,071	2.5
93	Tinea unguium	B351	12,949	2.5
94	Preretinal membrane	H353	12,892	2.4
95	Sensorineural hearing loss	H905	12,865	2.4
96	Renal disease	N289	12,446	2.4
97	Congestive heart failure	I500	12,195	2.3
98	Unspecified diabetic nephropathy	E142	12,170	2.3
99	Chronic obstructive pulmonary disease	J449	11,848	2.2
100	Carotid atherosclerosis	I652	11,729	2.2
101	Chronic hepatitis	K739	11,633	2.2
102	Neurogenic bladder	N319	11,428	2.2
103	Hemorrhoids	K649	11,352	2.2
104	Macular degeneration	H353	11,308	2.1
105	Pruritus	L299	11,203	2.1
106	Pollinosis	J301	11,001	2.1
107	Acute dermatitis	L309	10,954	2.1
108	Osteoarthrosis	M1999	10,889	2.1
109	Emphysema	J439	10,798	2.0
110	Calculus of kidney	N200	10,462	2.0
111	Atherosclerosis	I709	10,267	1.9
112	Depressive episode	F329	10,169	1.9
113	Seborrheic keratosis	L82	10,123	1.9
114	Trichiasis	H020	10,117	1.9
115	Spondylosis deformans	M4799	10,071	1.9
116	Chronic dermatitis	L309	9,963	1.9
117	Blepharitis	H010	9,853	1.9

118	Laryngopharyngitis	J060	9,776	1.9
119	lumbar disc herniation	M512	9,746	1.8
120	Ringworm	B359	9,634	1.8
121	Neurotic disorder	F489	9,621	1.8
122	Sleep apnea	G473	9,528	1.8
123	Gallbladder polyp	K828	9,264	1.8
124	Irritable bowel syndrome	K589	9,250	1.8
125	lumbar disc disorder	M519	9,194	1.7
126	Premature ventricular contraction	I493	9,125	1.7
127	Stomatitis	K121	9,094	1.7
128	Sciatica	M5438	8,873	1.7
129	Normal tension glaucoma	H401	8,687	1.6
130	Gastric cancer	C169	8,642	1.6
131	Tonsillitis	J039	8,389	1.6
132	Old myocardial infarction	I252	8,372	1.6
133	Helicobacter pylori gastritis	K296	8,262	1.6
134	Atopic dermatitis	L209	8,241	1.6
135	Type 2 diabetes mellitus without complications	E119	7,772	1.5
136	Urinary tract infection	N390	7,766	1.5
137	Mitral valve insufficiency	I340	7,740	1.5
138	Cyst of liver	K768	7,673	1.5
139	Posterior vitreous detachment	H438	7,514	1.4
140	Sequelae of cerebral infarction	I693	7,486	1.4
141	Myopia	H521	7,477	1.4
142	Corneal ulcer	H160	7,294	1.4
143	Cystitis	N309	7,286	1.4
144	Otitis externa	H609	7,263	1.4
145	Liver disease	K769	7,202	1.4
146	Ocular hypertension	H400	7,041	1.3
147	Tinea pedis	B351	7,029	1.3
148	Hiatus hernia	K449	6,993	1.3
149	Age-related macular degeneration	H353	6,918	1.3
150	Rheumatoid arthritis	M0690	6,903	1.3
151	Astigmatism	H522	6,791	1.3
152	Erosive gastritis	K296	6,744	1.3
153	Angina of effort	I208	6,741	1.3
154	Peripheral neuritis	G629	6,681	1.3
155	Conjunctival hemorrhage	H113	6,620	1.3
156	Influenza, virus not identified	J111	6,608	1.3
157	Ischemic heart disease	I259	6,475	1.2

158	Chronic kidney disease	N189	6,452	1.2
159	Cervical spondylotic radiculopathy	M4722	6,433	1.2
160	Pneumonia	J189	6,430	1.2
161	Hypothyroidism	E039	6,401	1.2
162	Inguinal hernia	K409	6,240	1.2
163	Calculus of ureter	N201	6,173	1.2
164	Chronic kidney failure	N189	6,144	1.2
165	Chronic prostatitis	N411	6,121	1.2
166	Arteriosclerotic retinopathy	I708	6,110	1.2
167	Hypermetropia	H520	5,988	1.1
168	Otitis externa eczematoid	H605	5,972	1.1
169	Coxarthrosis	M169	5,896	1.1
170	Acute tonsillitis	J039	5,878	1.1
171	Meniere's disease	H810	5,772	1.1
172	Obstruction of Eustachian tube	H681	5,752	1.1
173	Duodenal ulcer	K269	5,729	1.1
174	Respiratory failure	J9699	5,659	1.1
175	Xerosis cutis	L853	5,568	1.1
176	Tinnitus	H931	5,554	1.1
177	Arteriosclerotic fundus	H350	5,542	1.1
178	Allergic contact dermatitis	L239	5,524	1.0
179	Sinusitis	J329	5,455	1.0
180	Paroxysmal atrial fibrillation	I480	5,361	1.0
181	After-cataract	H264	5,342	1.0
182	Acute conjunctivitis	H103	5,306	1.0

The 100 most-diagnosed diseases (or those with >1% prevalence) among men aged 70-74 years are described. among men aged 40-44 years

ICD-10, International Classification of Diseases, 10th Revision.

Table S15. Prevalence of diagnosed diseases among women aged 40-44 years

Order	Names of Diagnoses	Corresponding ICD-10 Code	N	%
1	Allergic rhinitis	J304	281,173	38.4
2	Acute bronchitis	J209	164,343	22.4
3	Allergic conjunctivitis	H101	158,755	21.7
4	Astigmatism	H522	153,249	20.9
5	Acute upper respiratory infection	J069	123,478	16.8
6	Acute laryngopharyngitis	J060	112,934	15.4
7	Asthmatic bronchitis	J459	99,867	13.6
8	Acute sinusitis	J019	82,642	11.3
9	Chronic gastritis	K295	74,221	10.1
10	Acute pharyngitis	J029	68,426	9.33
11	Gastritis	K297	68,000	9.28
12	Eczema	L309	67,871	9.26
13	Low back pain	M5456	58,715	8.01
14	Iron deficiency anemia	D509	57,899	7.90
15	Leiomyoma of uterus	D259	55,853	7.62
16	Pharyngitis	J029	54,355	7.41
17	Acute gastritis	K291	51,086	6.97
18	Constipation	K590	48,922	6.67
19	influenza A	J101	47,409	6.47
20	Reflux esophagitis	K210	47,098	6.42
21	Primary ovarian failure	E283	47,019	6.41
22	Dry eye syndrome	H041	42,648	5.82
23	Asteatosis	L853	41,324	5.64
24	Sleep disorders	G470	40,467	5.52
25	Bronchitis	J40	39,398	5.37
26	Asteatotic eczema	L853	37,972	5.18
27	Atopic dermatitis	L209	32,233	4.40
28	Gastric ulcer	K259	32,226	4.40
29	Conjunctivitis	H109	31,932	4.36
30	Common cold	J00	31,397	4.28
31	Chronic sinusitis	J329	31,295	4.27
32	Urticaria	L509	30,988	4.23
33	Diarrhea	A099	28,374	3.87
34	Disorder of peripheral nervous system	G629	24,318	3.32
35	Hypertension	I10	24,158	3.30
36	Influenza, virus not identified	J111	24,100	3.29
37	Local infection of skin	L089	24,003	3.27

38	Tonsillitis	J039	23,001	3.14
39	Disorder of breast	N649	22,710	3.10
40	Ovarian cyst	D27	22,173	3.02
41	Myopia	H521	21,978	3.00
42	Keratoconjunctivitis sicca	H168	21,948	2.99
43	Migraine	G439	21,741	2.97
44	Endometriosis	N809	21,710	2.96
45	Laryngopharyngitis	J060	21,446	2.93
46	Acute tonsillitis	J039	20,618	2.81
47	Acne vulgaris	L700	20,404	2.78
48	Post inflammatory hyperpigmentation	L810	20,100	2.74
49	Contact dermatitis	L259	19,927	2.72
50	Depressive episode	F329	19,334	2.64
51	Dermatitis	L309	18,945	2.58
52	Acute dermatitis	L309	18,133	2.47
53	Dehydration	E86	17,797	2.43
54	Blepharitis	H010	17,432	2.38
55	Acute laryngitis	J040	16,949	2.31
56	Hyperlipidemia	E785	16,675	2.27
57	Presbyopia	H524	16,196	2.21
58	Neurotic disorder	F489	15,921	2.17
59	Cystitis	N309	15,911	2.17
60	Seborrheic dermatitis	L219	15,891	2.17
61	Lump in breast	N63	15,602	2.13
62	Pollinosis	J301	15,528	2.12
63	Anxiety neurosis	F411	15,507	2.12
64	Asthenopia	H531	15,112	2.06
65	Liver dysfunction	K769	14,675	2.00
66	Unspecified diabetes mellitus	E14	14,649	2.00
67	Enlarged optic nerve head excavation	H400	14,077	1.92
68	Sinusitis	J329	13,160	1.80
69	High myopia	H521	13,121	1.79
70	Anemia	D649	13,099	1.79
71	Pruritus	L299	12,287	1.68
72	Stomatitis	K121	12,171	1.66
73	Abnormal uterine and vaginal bleeding	N939	12,003	1.64
74	Dyslipidemia	E785	11,584	1.58
75	Intractable reflux esophagitis with maintenance therapy	K210	11,105	1.51
76	Menopausal disorder	N951	10,885	1.48
77	Ovarian neoplasm	D391	10,830	1.48

78	Chronic dermatitis	L309	10,777	1.47
79	Hypothyroidism	E039	10,540	1.44
80	Hypercholesterolemia	E780	10,531	1.44
81	lumbar disc disorder	M519	10,527	1.44
82	Hypermetropic astigmatism	H522	10,460	1.43
83	Glaucoma	H409	10,422	1.42
84	Tinea pedis	B353	10,399	1.42
85	lumbar disc herniation	M512	10,221	1.39
86	Impacted cerumen	H612	10,163	1.39
87	Irritable bowel syndrome	K589	10,127	1.38
88	Otitis externa	H609	10,077	1.37
89	Chronic bronchitis	J42	10,046	1.37
90	Otitis externa eczematoid	H605	9,828	1.34
91	Acute rhinitis	J00	9,760	1.33
92	Acute conjunctivitis	H103	9,637	1.31
93	Internal hemorrhoid	K649	9,357	1.28
94	Gonarthrosis	M171	9,349	1.28
95	Meniere's disease	H810	9,153	1.25
96	Superficial punctate keratitis	H161	9,129	1.25
97	Atrophic gastritis	K294	9,110	1.24
98	Hordeolum	H000	9,016	1.23
99	Myalgia	M7919	8,853	1.21
100	Asthmatic bronchitis	J459	8,844	1.21
101	Candidal vulvovaginitis	B373	8,835	1.21
102	Sensorineural hearing loss	H905	8,649	1.18
103	Acute exacerbation of chronic sinusitis	J329	8,560	1.17
104	Helicobacter pylori infection	A498	8,539	1.16
105	Schizophrenia	F209	8,538	1.16
106	Hand eczema	L309	8,420	1.15
107	Breast neoplasm	D486	8,281	1.13
108	Cervical spondylosis	M4782	8,217	1.12
109	Polyp of stomach	K317	8,103	1.11
110	Hashimoto thyroiditis	E063	7,983	1.09
111	Xerosis cutis	L853	7,925	1.08
112	Luteal phase deficiency	E283	7,905	1.08
113	Corneal ulcer	H160	7,896	1.08
114	Depressive episode	F329	7,587	1.03
115	Obstruction of Eustachian tube	H681	7,563	1.03
116	Hyperprolactinemia	E221	7,463	1.02

The 100 most-diagnosed diseases (or those with >1% prevalence) among men aged 40-44 years are described.

among women aged 40-44 years

ICD-10, International Classification of Diseases, 10th Revision.

Table S16. Prevalence of diagnosed diseases among women aged 70-74 years

Order	Names of Diagnoses	Corresponding ICD-10 Code	N	%
1	Hypertension	I10	282,969	44.9
2	Allergic rhinitis	J304	212,204	33.7
3	Osteoporosis	M8199	171,382	27.2
4	Hyperlipidemia	E785	153,349	24.3
5	Chronic gastritis	K295	151,986	24.1
6	Hypercholesterolemia	E780	150,920	23.9
7	Allergic conjunctivitis	H101	144,506	22.9
8	Cataract	H269	142,760	22.6
9	Sleep disorders	G470	137,843	21.9
10	Hypermetropic astigmatism	H522	126,176	20.0
11	Gonarthrosis	M171	120,802	19.2
12	Astigmatism	H522	119,953	19.0
13	Low back pain	M5456	119,141	18.9
14	Acute bronchitis	J209	114,829	18.2
15	Constipation	K590	113,135	17.9
16	Unspecified diabetes mellitus	E14	109,135	17.3
17	Reflux esophagitis	K210	107,708	17.1
18	Dry eye syndrome	H041	91,920	14.6
19	Gastritis	K297	91,679	14.5
20	Acute upper respiratory infection	J069	90,662	14.4
21	Dyslipidemia	E785	80,308	12.7
22	Eczema	L309	79,978	12.7
23	Gastric ulcer	K259	76,801	12.2
24	Asthmatic bronchitis	J459	76,493	12.1
25	Disorder of peripheral nervous system	G629	64,363	10.2
26	Conjunctivitis	H109	59,796	9.48
27	Lumbar spondylosis deformans	M4786	56,977	9.04
28	Presbyopia	H524	56,480	8.96
29	Acute laryngopharyngitis	J060	56,311	8.93
30	Intractable reflux esophagitis with maintenance therapy	K210	55,846	8.86
31	Lumbar spinal stenosis	M4806	55,543	8.81
32	Asthenopia	H531	49,213	7.81
33	Keratoconjunctivitis sicca	H168	49,181	7.80
34	Glaucoma	H409	46,585	7.39
35	Angina pectoris	I209	45,595	7.23
36	Pharyngitis	J029	42,798	6.79
37	Acute pharyngitis	J029	41,682	6.61

38	Asteatosis	L853	39,935	6.33
39	Anxiety neurosis	F411	39,416	6.25
40	Atrophic gastritis	K294	38,636	6.13
41	Type 2 diabetes mellitus	E11	38,327	6.08
42	Fatty liver	K760	38,066	6.04
43	Asteatotic eczema	L853	37,218	5.90
44	Senile cataract	H259	35,043	5.56
45	Acute sinusitis	J019	35,033	5.56
46	Liver disease	K769	34,373	5.45
47	Bronchitis	J40	32,696	5.19
48	Acute gastritis	K291	30,023	4.76
49	Polyp of colon	K635	29,846	4.73
50	Tinea pedis	B353	29,119	4.62
51	Enlarged optic nerve head excavation	H400	28,643	4.54
52	Mixed astigmatism	H522	28,543	4.53
53	Cystitis	N309	28,479	4.52
54	Common cold	J00	28,444	4.51
55	Blepharitis	H010	27,991	4.44
56	Overactive bladder	N328	26,682	4.23
57	Cardiac arrhythmia	I499	26,325	4.18
58	Intractable reflux esophagitis	K210	25,919	4.11
59	Cervical spondylosis deformans	M4782	25,903	4.11
60	Myalgia	M7919	25,764	4.09
61	Contact dermatitis	L259	25,260	4.01
62	Dermatitis	L309	24,916	3.95
63	Chronic bronchitis	J42	24,754	3.93
64	Iron deficiency anemia	D509	24,701	3.92
65	Chronic sinusitis	J329	24,477	3.88
66	Neuropathic pain	G98	24,073	3.82
67	Dehydration	E86	23,794	3.77
68	Trichiasis	H020	23,548	3.73
69	Depressive episode	F329	23,038	3.65
70	Impacted cerumen	H612	22,960	3.64
71	Helicobacter pylori infection	A498	22,555	3.58
72	Cervical spondylosis	M4782	22,024	3.49
73	Urticaria	L509	21,983	3.49
74	Polyp of stomach	K317	21,851	3.47
75	Neurotic disorder	F489	21,302	3.38
76	Diarrhea	A099	21,220	3.37
77	Stomatitis	K121	20,698	3.28

78	Coxarthrosis	M169	20,656	3.28
79	Preretinal membrane	H353	20,634	3.27
80	Arteriosclerosis obliterans	I709	20,207	3.20
81	Osteoarthrosis	M1999	20,114	3.19
82	Peripheral neuropathic pain	G64	19,945	3.16
83	Cerebral infarction	I639	19,561	3.10
84	Sensorineural hearing loss	H905	19,299	3.06
85	Local infection of skin	L089	18,893	3.00
86	Spondylosis deformans	M4799	18,888	3.00
87	Rheumatoid arthritis	M0690	18,226	2.89
88	Acute dermatitis	L309	18,113	2.87
89	Heart failure	I509	17,761	2.82
90	influenza A	J101	17,685	2.80
91	Hypothyroidism	E039	17,478	2.77
92	Essential hypertension	I10	17,348	2.75
93	Hyperuricemia	E790	17,077	2.71
94	Calculus of gallbladder	K802	16,125	2.56
95	Unspecified diabetes retinopathy	E143	16,016	2.54
96	Pollinosis	J301	16,003	2.54
97	Macular degeneration	H353	15,739	2.50
98	Chronic heart failure	I509	15,631	2.48
99	Carotid atherosclerosis	I652	15,617	2.48
100	lumbar disc disorder	M519	15,597	2.47
101	Meniere’s disease	H810	15,404	2.44
102	Internal hemorrhoid	K649	15,257	2.42
103	Pruritus	L299	15,228	2.42
104	Carotid artery stenosis	I652	15,106	2.40
105	Sciatica	M5438	14,965	2.37
106	Laryngopharyngitis	J060	14,752	2.34
107	Anemia	D649	14,693	2.33
108	Malignant neoplasm of breast	C509	14,346	2.28
109	Seborrheic dermatitis	L219	14,342	2.27
110	Atherosclerosis	I709	14,181	2.25
111	Tinea unguium	B351	14,155	2.25
112	Corneal ulcer	H160	14,118	2.24
113	Posterior vitreous detachment	H438	13,941	2.21
114	Irritable bowel syndrome	K589	13,911	2.21
115	Atopic dermatitis	L209	13,407	2.13
116	Postmenopausal osteoporosis	M8109	13,277	2.11
117	Tonsillitis	J039	13,193	2.09

118	Cyst of liver	K768	12,889	2.04
119	Hypermetropia	H520	12,848	2.04
120	Spondylolisthesis	M4316	12,666	2.01
121	Normal tension glaucoma	H401	12,184	1.93
122	Seborrheic keratosis	L82	12,132	1.92
123	Disorder of breast	N649	12,088	1.92
124	Arteriosclerotic retinopathy	I708	12,020	1.91
125	Stiff neck	M6281	11,958	1.90
126	Peripheral neuritis	G629	11,896	1.89
127	Hemorrhoids	K649	11,760	1.87
128	Chronic dermatitis	L309	11,671	1.85
129	Leiomyoma of uterus	D259	11,410	1.81
130	Diffuse superficial keratitis	H161	11,390	1.81
131	lumbar disc herniation	M512	11,337	1.80
132	Goiter	E049	11,219	1.78
133	Renal disease	N289	11,110	1.76
134	Conjunctival hemorrhage	H113	11,024	1.75
135	Chronic hepatitis	K739	10,971	1.74
136	Superficial punctate keratitis	H161	10,873	1.72
137	Cyst of kidney	N281	10,831	1.72
138	Dermatitis of eyelid	H011	10,772	1.71
139	Peripheral vascular disease	I739	10,729	1.70
140	Otitis externa	H609	10,667	1.69
141	Gallbladder polyp	K828	10,573	1.68
142	Myopia	H521	10,515	1.67
143	Arteriosclerotic fundus	H350	10,451	1.66
144	Obstruction of Eustachian tube	H681	10,376	1.65
145	Astigmatism	H522	10,222	1.62
146	Corns	L84	10,209	1.62
147	Otitis externa eczematoid	H605	10,092	1.60
148	Disorder of autonomic nervous system	G909	10,062	1.60
149	Helicobacter pylori gastritis	K296	9,750	1.55
150	Ocular hypertension	H400	9,609	1.52
151	Psychophysiological disorder	F459	9,546	1.51
152	Hashimoto thyroiditis	E063	9,516	1.51
153	After-cataract	H264	9,436	1.50
154	Congestive heart failure	I500	9,394	1.49
155	Vitreous opacity	H433	9,275	1.47
156	Acute tonsillitis	J039	9,036	1.43
157	Keratitis	H169	8,997	1.43

158	Allergic contact dermatitis	L239	8,939	1.42
159	Neurogenic bladder	N319	8,764	1.39
160	Myodesopsia	H438	8,738	1.39
161	Acute laryngitis	J040	8,722	1.38
162	Acute conjunctivitis	H103	8,616	1.37
163	Cervical spondylotic radiculopathy	M4722	8,580	1.36
164	Ringworm	B359	8,577	1.36
165	Premature ventricular contraction	I493	8,510	1.35
166	Influenza, virus not identified	J111	8,488	1.35
167	Tinnitus	H931	8,437	1.34
168	Xerosis cutis	L853	8,088	1.28
169	Migraine	G439	8,052	1.28
170	Mitral valve insufficiency	I340	8,045	1.28
171	Joint pain	M2559	7,926	1.26
172	Thyroid neoplasm	D440	7,878	1.25
173	Post inflammatory hyperpigmentation	L810	7,877	1.25
174	Atrial fibrillation	I489	7,874	1.25
175	Hyperthyroidism	E059	7,806	1.24
176	Sinusitis	J329	7,764	1.23
177	Urinary tract infection	N390	7,760	1.23
178	Chronic conjunctivitis	H104	7,742	1.23
179	Erosive gastritis	K296	7,705	1.22
180	Arthritis	M1399	7,590	1.20
181	Hypertensive retinopathy	H350	7,535	1.20
182	Sjogren's syndrome	M350	7,518	1.19
183	Unspecified diabetic nephropathy	E142	7,484	1.19
184	Depressive episode	F329	7,477	1.19
185	Calculus of kidney	N200	7,401	1.17
186	Knee joint pain	M2556	7,370	1.17
187	Nerve pain	M7929	7,285	1.16
188	Age-related macular degeneration	H353	7,223	1.15
189	Serous otitis media	H659	7,215	1.14
190	Liver dysfunction	K769	7,214	1.14
191	Varicose vein	I839	7,201	1.14
192	Hiatus hernia	K449	7,028	1.11
193	Tinea pedis	B351	6,981	1.11
194	Nontoxic multinodular goiter	E049	6,794	1.08
195	Palmoplantar keratoderma	L851	6,758	1.07
196	Hip joint pain	M2555	6,735	1.07
197	Chorioretinal atrophy	H311	6,661	1.06

198	Marginal blepharitis	H010	6,645	1.05
199	Tenosynovitis	M6599	6,590	1.05
200	Lump in breast	N63	6,559	1.04
201	Herpetic keratitis	H161	6,448	1.02
202	Postoperative iritis	H598	6,418	1.02
203	Hallux valgus	M201	6,418	1.02
204	Asthmatic bronchitis	J459	6,381	1.01
205	Hearing loss	H919	6,349	1.01

The 100 most-diagnosed diseases (or those with >1% prevalence) among men aged 40-44 years are described.
among women aged 70-74 years

ICD-10, International Classification of Diseases, 10th Revision.

Table S17. Prevalence of prescribed medications among men aged 40-44 years

Order	Therapeutic Category	Drug name	N	%	number of generic drugs
1	Antipyretics and analgesics, anti-inflammatory agents	Loxoprofen Sodium Hydrate 60mg generic	175,862	17.3	17
2	Peptic ulcer agents	Rebamipide 100mg generic	142,025	14.0	28
3	Expectorants	L-Carbocisteine 500mg generic	140,352	13.8	4
4	Hemostatics	Tranexamic Acid 250mg generic	91,440	9.01	3
5	Antipyretics and analgesics, anti-inflammatory agents	Acetaminophen 200mg original	91,048	8.97	—
6	Acting mainly on gram-positive bacteria and mycoplasma	Clarithromycin 200mg generic	64,672	6.37	14
7	Antitussives	Dextromethorphan Hydrobromide Hydrate 15mg generic	60,959	6.01	2
8	Other allergic agents	Montelukast Sodium 10mg generic	55,944	5.51	38
9	Common cold drugs	Salicylamide/Acetaminophen/Anhydrous Caffeine/ Promethazine Methylenedisalicylate original	54,786	5.40	—
10	Other allergic agents	Fexofenadine Hydrochloride 60mg generic	53,029	5.23	5
11	Acting mainly on gram-positive and gram-negative bacteria	Cefcapene Pivoxil Hydrochloride Hydrate 100mg generic	52,407	5.16	5
12	Antitussives	Dihydrocodeine Phosphate/dl-Methylephedrine Hydrochloride/ Chlorpheniramine Maleate original	43,236	4.26	—
13	Acting mainly on gram-positive and gram-negative bacteria	Cefditoren Pivoxil 100mg generic	42,869	4.22	5
14	Antipyretics and analgesics, anti-inflammatory agents	Acetaminophen 500mg original	40,763	4.02	—
15	Other allergic agents	Bilastine 20mg original	40,069	3.95	—
16	Antipyretics and analgesics, anti-inflammatory agents	Acetaminophen 300mg original	38,668	3.81	—
17	Expectorants	L-Carbocisteine 250mg generic	37,666	3.71	2
18	Antipyretics and analgesics, anti-inflammatory agents	Loxoprofen Sodium Hydrate 60mg original	37,000	3.65	—
19	Other allergic agents	Levocetirizine Hydrochloride 5mg original	36,261	3.57	—
20	Antitussives and expectorants	Tipepidine Hibenzate 20mg original	35,058	3.45	—
21	Other digestive organ agents	Dequalinium Chloride Troches 0.25mg generic	34,107	3.36	1
22	Ophthalmic agents	Olopatadine Hydrochloride Solution 0.1% original	32,312	3.18	—

23	Synthetic antibacterials	Levofloxacin Hydrate 500mg generic	32,221	3.17	30
24	Synthetic antibacterials	Garenoxacin Mesilate Hydrate 200mg original	32,218	3.17	—
25	Otic and nasal agents	Fluticasone Furoate 27.5μg metered Nasal Spray original	31,201	3.07	—
26	Antivirals	Baloxavir Marboxil 20mg original	30,218	2.98	—
27	Hemostatics	Tranexamic Acid 500mg generic	30,150	2.97	1
28	Antidiarrheals, intestinal regulators	Clostridium butyricum original	30,028	2.96	—
29	Other allergic agents	Bepotastine Besilate 10mg generic	28,907	2.85	13
30	Antitussives	Dextromethorphan Hydrobromide Hydrate 15mg original	27,213	2.68	—
31	Expectorants	L-Carbocisteine 500mg original	26,628	2.62	—
32	Antidiarrheals, intestinal regulators	Antibiotics-Resistant Lactic Acid Bacteriae original	25,543	2.52	—
33	Acting mainly on gram-positive and gram-negative bacteria	Amoxicillin Hydrate 250mg generic	20,199	1.99	2
34	Other allergic agents	Desloratadine 5mg original	18,577	1.83	—
35	Antivirals	Laninamivir Octanoate Hydrate Inhaler 20mg original	18,512	1.82	—
36	Hemostatics	Tranexamic Acid 250mg original	18,099	1.78	—
37	Otic and nasal agents	Mometasone Furoate Hydrate Nasal 50μg 56 sprays original	17,753	1.75	—
38	Peptic ulcer agents	Rebamipide 100mg original	17,504	1.72	—
39	Antidiarrheals, intestinal regulators	Enterococcus faecium/ Clostridium butyricum/Bacillus subtilis original	16,256	1.60	—
40	Antitussives	Dimemorfan Phosphate 10mg original	16,081	1.58	—
41	Antipyretics and analgesics, anti-inflammatory agents	Celecoxib 100mg original	15,929	1.57	—
42	Analgesics, anti-itchings, astringents, anti-inflammatory agents	Betamethasone Butyrate Propionate Ointment 0.05% original	15,874	1.56	—
43	Acting mainly on gram-positive and gram-negative bacteria	Cefcapene Pivoxil Hydrochloride Hydrate 100mg original	15,518	1.53	—
44	Antitussives	Diprophylline/Dihydrocodeine Phosphate/dl-Methylephedrine Hydrochloride/ Diphenhydramine Salicylate/Acetaminophen/Bromovalerylurea original	14,742	1.45	—
45	Analgesics, anti-itchings, astringents, anti-inflammatory agents	Betamethasone Valerate/Gentamicin Sulfate Ointment 0.12% original	14,492	1.43	—
46	Antidiarrheals, intestinal regulators	Bifidobacterium original	14,141	1.39	—

47	Acting mainly on gram-positive bacteria and mycoplasma	Clarithromycin 200mg original	14,096	1.39	—
48	Peptic ulcer agents	Esomeprazole Magnesium Hydrate 20mg original	13,982	1.38	—
49	Anticoagulants	Heparinoid Ointment 0.3% original	13,894	1.37	—
50	Gout preparations	Febuxostat 10mg original	13,661	1.35	—

The 50 most-prescribed medications are described according to their therapeutic category, nonproprietary names, whether they are original or generic products, and their dosage.

Therapeutic categories were allocated according to the package insert or the Prescription Medications in Pharmaceuticals and Medical Devices Agency's search system [20].

Table S18. Prevalence of prescribed medications among men aged 70-74 years

Order	Therapeutic Category	Drug name	N	%	number of generic drugs
1	Peptic ulcer agents	Rebamipide 100mg generic	77,831	14.7	28
2	Antipyretics and analgesics, anti-inflammatory agents	Loxoprofen Sodium Hydrate 60mg generic	73,180	13.9	17
3	Expectorants	L-Carbocisteine 500mg generic	45,047	8.54	2
4	Analgesics, anti-itchings, astringents, anti-inflammatory agents	Loxoprofen Sodium Hydrate Tape/ Patch 100mg generic	33,929	6.43	30
5	Antipyretics and analgesics, anti-inflammatory agents	Acetaminophen 200mg original	32,660	6.19	—
6	Other blood and body fluid agents	Aspirin 100mg original	30,739	5.82	—
7	Hemostatics	Tranexamic Acid 250mg generic	29,172	5.53	3
8	Hyperlipidemia agents	Rosuvastatin Calcium 2.5mg generic	28,817	5.46	40
9	Antitussives	Dextromethorphan Hydrobromide Hydrate 15mg generic	26,626	5.05	2
10	Acting mainly on gram-positive and gram-negative bacteria	Cefcapene Pivoxil Hydrochloride Hydrate 100mg generic	26,482	5.02	5
11	Common cold drugs	Salicylamide/Acetaminophen/ Anhydrous Caffeine/ Promethazine Methylenedisalicylate original	26,452	5.01	3
12	Gout preparations	Allopurinol 100mg generic	23,842	4.52	15
13	Antipyretics and analgesics, anti-inflammatory agents	Celecoxib 100mg original	23,202	4.40	—
14	Peptic ulcer agents	Esomeprazole Magnesium Hydrate 20mg original	22,040	4.18	—
15	Gout preparations	Febuxostat 10mg original	20,857	3.95	—
16	Ophthalmic agents	Olopatadine Hydrochloride Solution 0.1% original	20,304	3.85	—
17	Other allergic agents	Montelukast Sodium 10mg generic	19,594	3.71	36
18	Other urogenital and anal organ agents	Silodosin 4mg original	19,246	3.65	2
19	Expectorants	L-Carbocisteine 250mg generic	19,180	3.63	2
20	Antipyretics and analgesics, anti-inflammatory agents	Loxoprofen Sodium Hydrate 60mg original	18,106	3.43	—
21	Vitamin B preparations	Mecobalamin 500μg original	17,134	3.25	—
22	Other allergic agents	Levocetirizine Hydrochloride 5mg original	16,229	3.08	—
23	Anticoagulants	Heparinoid Cream/ Lotion/ Spray/ Gel 0.3% generic	16,213	3.07	23

24	Analgesics, anti-itchings, astringents, anti-inflammatory agents	Ketoprofen Tapes 40mg original	16,119	3.05	—
25	Other allergic agents	Bilastine 20mg original	15,991	3.03	—
26	Antitussives	Dextromethorphan Hydrobromide Hydrate 15mg original	15,455	2.93	—
27	Other digestive organ agents	Dequalinium Chloride 25mg generic	15,048	2.85	1
28	Analgesics, anti-itchings, astringents, anti-inflammatory agents	Betamethasone Valerate/Gentamicin Sulfate Ointment 0.12% original	14,709	2.79	
29	Antitussives and expectorants	Tipepidine Hibenzate 20mg original	14,547	2.76	—
30	Gout preparations	Febuxostat 20mg original	14,234	2.70	—
31	Antipyretics and analgesics, anti-inflammatory agents	Acetaminophen 300mg original	14,042	2.66	—
32	Antacids	Magnesium Oxide 330mg generic	14,025	2.66	4
33	Analgesics, anti-itchings, astringents, anti-inflammatory agents	Betamethasone Butyrate Propionate Ointment 0.05% original	13,390	2.54	—
34	Otic and nasal agents	Fluticasone Furoate 27.5μg metered Nasal Spray original	13,224	2.51	—
35	Anticoagulants	Heparinoid Ointment 0.3% original	12,874	2.44	—
36	Analgesics, anti-itchings, astringents, anti-inflammatory agents	Loxoprofen Sodium Hydrate Tapes 100mg original	12,491	2.37	—
37	Antipyretics and analgesics, anti-inflammatory agents	Acetaminophen 500mg original	12,336	2.34	—
38	Antitussives	Dihydrocodeine Phosphate/dl-Methylephedrine Hydrochloride/ Chlorpheniramine Maleate original	12,273	2.33	—
39	Peptic ulcer agents	Vonoprazan Fumarate 10mg original	12,003	2.27	—
40	Expectorants	L-Carbocisteine 500mg original	11,824	2.24	—
41	Antidiarrheals, intestinal regulators	Clostridium butyricum original	11,665	2.21	—
42	Antidiabetic agents	Sitagliptin Phosphate Hydrate 50mg original	11,184	2.12	—
43	Other hormone preparations	Dutasteride 5mg original	11,069	2.10	—
44	Peptic ulcer agents	Rebamipide 100mg original	10,945	2.07	—
45	Otic and nasal agents	Mometasone Furoate Hydrate Nasal 50μg 56 sprays original	9,774	1.85	—
46	Antidiabetic agents	Metformin Hydrochloride 250mg original	9,341	1.77	—
47	Acting mainly on gram-positive and gram-negative bacteria	Cefcapene Pivoxil Hydrochloride Hydrate 100mg original	9,317	1.77	—

48	Other agents affecting central nervous system	Pregabalin 25mg original	9,229	1.75	—
49	Peptic ulcer agents	Vonoprazan Fumarate 20mg original	9,149	1.73	—
50	Antacids	Magnesium Oxide 330mg original	9,068	1.72	—

The 50 most-prescribed medications are described according to their therapeutic category, nonproprietary names, whether they are original or generic products, and their dosage.

Therapeutic categories were allocated according to the package insert or the Prescription Medications in Pharmaceuticals and Medical Devices Agency's search system [20].

Table S19. Prevalence of prescribed medications among women aged 40-44 years

Order	Therapeutic Category	Drug name	N	%	number of generic drugs
1	Expectorants	L-Carbocisteine 500mg generic	137,249	18.7	5
2	Antipyretics and analgesics, anti-inflammatory agents	Loxoprofen Sodium Hydrate 60mg generic	133,286	18.2	18
3	Peptic ulcer agents	Rebamipide 100mg generic	117,057	16.0	28
4	Hemostatics	Tranexamic Acid 250mg generic	96,028	13.1	3
5	Antipyretics and analgesics, anti-inflammatory agents	Acetaminophen 200mg original	86,716	11.8	—
6	Acting mainly on gram-positive bacteria and mycoplasma	Clarithromycin 200mg generic	68,963	9.41	14
7	Other allergic agents	Montelukast Sodium 10mg generic	68,477	9.34	37
8	Other allergic agents	Fexofenadine Hydrochloride 60mg generic	59,702	8.14	31
9	Antitussives	Dextromethorphan Hydrobromide Hydrate generic	58,335	7.96	2
10	Expectorants	L-Carbocisteine 250mg generic	52,845	7.21	5
11	Other allergic agents	Bilastine 20mg original	48,922	6.67	—
12	Acting mainly on gram-positive and gram-negative bacteria	Cefcapene Pivoxil Hydrochloride Hydrate 100mg generic	46,805	6.38	5
13	Acting mainly on gram-positive and gram-negative bacteria	Cefditoren Pivoxil 100mg generic	44,078	6.01	5
14	Antitussives	Dihydrocodeine Phosphate/dl-Methylephedrine Hydrochloride/Chlorpheniramine Maleate original	43,261	5.90	—
15	Other allergic agents	Levocetirizine Hydrochloride 5mg original	42,955	5.86	—
16	Common cold drugs	Salicylamide/Acetaminophen/Anhydrous Caffeine/ Promethazine Methylenedisalicylate original	42,403	5.78	3
17	Ophthalmic agents	Olopatadine Hydrochloride Solution 0.1% original	41,639	5.68	—
18	Antitussives and expectorants	Tipepidine Hibenzate 20mg original	39,584	5.40	—
19	Antipyretics and analgesics, anti-inflammatory agents	Loxoprofen Sodium Hydrate 60mg original	39,508	5.39	—
20	Other digestive organ agents	Dequalinium Chloride Troches 0.25mg generic	37,764	5.15	—
21	Otic and nasal agents	Fluticasone Furoate 27.5µg metered Nasal Spray original	36,293	4.95	—
22	Expectorants	L-Carbocisteine 500mg original	34,784	4.74	—
23	Antipyretics and analgesics, anti-inflammatory agents	Acetaminophen 300mg original	34,744	4.74	—

24	Other allergic agents	Bepotastine Besilate 10mg generic	34,040	4.64	13
25	Antitussives	Dextromethorphan Hydrobromide Hydrate 15mg original	31,602	4.31	—
26	Anticoagulants	Heparinoid Cream/ Lotion/ Spray/ Gel 0.3% generic	29,353	4.00	25
27	Synthetic antibacterials	Garenoxacin Mesilate Hydrate 200mg original	28,956	3.95	—
28	Antidiarrheals, intestinal regulators	Antibiotics-Resistant Lactic Acid Bacteriae original	28,315	3.86	—
29	Antipyretics and analgesics, anti-inflammatory agents	Acetaminophen 500mg original	28,271	3.86	—
30	Antidiarrheals, intestinal regulators	Clostridium butyricum original	27,957	3.81	—
31	Hemostatics	Tranexamic Acid 500mg generic	27,096	3.70	1
32	Antivirals	Baloxavir Marboxil 20mg original	25,688	3.50	—
33	Anticoagulants	Heparinoid Ointment 0.3% original	23,717	3.24	—
34	Hemostatics	Tranexamic Acid 250mg original	23,473	3.20	—
35	Other allergic agents	Desloratadine 5mg original	22,585	3.08	—
36	Peptic ulcer agents	Rebamipide 100mg original	20,967	2.86	—
37	Analgesics, anti-itchings, astringents, anti-inflammatory agents	Betamethasone Butyrate Propionate Ointment 0.05% original	19,581	2.67	—
38	Acting mainly on gram-positive bacteria and mycoplasma	Clarithromycin 200mg original	19,436	2.65	—
39	Otic and nasal agents	Mometasone Furoate Hydrate Nasal 50µg 56 sprays original	18,583	2.53	—
40	Multivitamin preparations	Ascorbic Acid/Calcium/Pantothenate original	18,440	2.52	—
41	Analgesics, anti-itchings, astringents, anti-inflammatory agents	Betamethasone Valerate/Gentamicin Sulfate Ointment 0.12% original	18,113	2.47	—
42	Analgesics, anti-itchings, astringents, anti-inflammatory agents	Hydrocortisone Butyrate Ointment 0.1% original	17,760	2.42	—
43	Acting mainly on gram-positive and gram-negative bacteria	Cefcapene Pivoxil Hydrochloride Hydrate 100mg original	17,531	2.39	—
44	Antitussives	Dimemorfan Phosphate 10mg original	16,987	2.32	—
45	Antivirals	Laninamivir Octanoate Hydrate Inhaler 20mg original	16,571	2.26	—
46	Anticoagulants	Heparinoid Lotion 0.3% original	16,443	2.24	—
47	Acting mainly on gram-positive and gram-negative bacteria	Cefditoren Pivoxil 100mg original	15,903	2.17	—
48	Other allergic agents	Rupatadine Fumarate 10mg original	15,896	2.17	—
49	Ophthalmic agents	Diquafosol Sodium Solution 3% original	15,682	2.14	—

50	Antidiarrheals, intestinal regulators	Enterococcus faecium/ Clostridium butyricum/Bacillus subtilis original	15,261	2.08	—

The 50 most-prescribed medications are described according to their therapeutic category, nonproprietary names, whether they are original or generic products, and their dosage.

Therapeutic categories were allocated according to the package insert or the Prescription Medications in Pharmaceuticals and Medical Devices Agency’s search system [20].

Table S20. Prevalence of prescribed medications among women aged 70-74 years

Order	Therapeutic Category	Drug name	N	%	number of generic drugs
1	Peptic ulcer agents	Rebamipide 100mg generic	116,240	18.4	28
2	Antipyretics and analgesics, anti-inflammatory agents	Loxoprofen Sodium Hydrate 60mg generic	90,351	14.3	18
3	Expectorants	L-Carbocisteine 500mg generic	69,730	11.1	5
4	Analgesics, anti-itchings, astringents, anti-inflammatory agents	Loxoprofen Sodium Hydrate Tape/ Patch 100mg generic	63,296	10.0	33
5	Vitamins A and D preparations	Eldecalcitol 0.75μg original	50,600	8.03	—
6	Antipyretics and analgesics, anti-inflammatory agents	Acetaminophen 200mg original	49,049	7.78	—
7	Antipyretics and analgesics, anti-inflammatory agents	Celecoxib 100mg original	47,009	7.46	—
8	Hyperlipidemia agents	Rosuvastatin Calcium 2.5mg generic	44,455	7.05	40
9	Common cold drugs	Salicylamide/Acetaminophen/Anhydrous Caffeine/ Promethazine Methylenedisalicylate original	44,404	7.04	3
10	Hemostatics	Tranexamic Acid 250mg generic	43,923	6.97	3
11	Ophthalmic agents	Olopatadine Hydrochloride Solution 0.1% original	37,277	5.91	—
12	Antitussives	Dextromethorphan Hydrobromide Hydrate 15mg generic	35,856	5.69	2
13	Acting mainly on gram-positive and gram-negative bacteria	Cefcapene Pivoxil Hydrochloride Hydrate 100mg generic	34,237	5.43	5
14	Expectorants	L-Carbocisteine 250mg generic	34,157	5.42	5
15	Antipyretics and analgesics, anti-inflammatory agents	Loxoprofen Sodium Hydrate 60mg original	32,638	5.18	—
16	Ophthalmic agents	Diquafosol Sodium Solution 3% original	30,562	4.85	—
17	Vitamin B preparations	Mecobalamin 500μg original	29,339	4.65	—
18	Analgesics, anti-itchings, astringents, anti-inflammatory agents	Ketoprofen Tapes 40mg original	29,079	4.61	—
19	Other allergic agents	Montelukast Sodium 10mg generic	28,315	4.49	37
20	Peptic ulcer agents	Esomeprazole Magnesium Hydrate 20mg original	27,375	4.34	—
21	Other digestive organ agents	Dequalinium Chloride Troches 0.25mg generic	27,268	4.32	—

22	Analgesics, anti-itchings, astringents, anti-inflammatory agents	Loxoprofen Sodium Hydrate Tapes 100mg original	26,249	4.16	—
23	Antitussives	Dextromethorphan Hydrobromide Hydrate 15mg original	25,369	4.02	—
24	Peptic ulcer agents	Rebamipide 100mg original	24,399	3.87	—
25	Other allergic agents	Bilastine 20mg original	23,959	3.80	—
26	Anticoagulants	Heparinoid Cream/ Lotion/ Spray/ Gel 0.3% generic	22,992	3.65	26
27	Expectorants	L-Carbocisteine 500mg original	22,807	3.62	—
28	Antitussives and expectorants	Tipepidine Hibenzate 20mg original	22,770	3.61	—
29	Analgesics, anti-itchings, astringents, anti-inflammatory agents	Betamethasone Valerate/Gentamicin Sulfate Ointment 0.12% original	22,357	3.55	—
30	Antipyretics and analgesics, anti-inflammatory agents	Acetaminophen 300mg original	22,051	3.50	—
31	Other allergic agents	Levocetirizine Hydrochloride 5mg original	21,822	3.46	—
32	Antitussives	Dihydrocodeine Phosphate/dl-Methylephedrine Hydrochloride/ Chlorpheniramine Maleate original	20,586	3.27	—
33	Antacids	Magnesium Oxide 330mg generic	20,533	3.26	4
34	Anticoagulants	Heparinoid Ointment 0.3% original	19,595	3.11	—
35	Antidiarrheals, intestinal regulators	Clostridium butyricum original	17,832	2.83	—
36	Other agents affecting metabolism	Adenosine Triphosphate Disodium Hydrate 10% original	16,746	2.66	—
37	Peptic ulcer agents	Vonoprazan Fumarate 10mg original	16,470	2.61	—
38	Acting mainly on gram-positive and gram-negative bacteria	Cefcapene Pivoxil Hydrochloride Hydrate 100mg original	16,306	2.59	—
39	Antipyretics and analgesics, anti-inflammatory agents	Acetaminophen 500mg original	15,556	2.47	—
40	Analgesics, anti-itchings, astringents, anti-inflammatory agents	Betamethasone Butyrate Propionate Ointment 0.05% original	15,409	2.44	—
41	Otic and nasal agents	Fluticasone Furoate 27.5µg metered Nasal Spray original	14,917	2.37	—
42	Other blood and body fluid agents	Aspirin 100mg original	14,873	2.36	—
43	Analgesics, anti-itchings, astringents, anti-inflammatory agents	Hydrocortisone Butyrate Ointment 0.1% original	14,512	2.30	—
44	Other agents affecting central nervous system	Pregabalin 25mg original	14,508	2.30	—

45	Analgesics, anti-itchings, astringents, anti-inflammatory agents	Ketoprofen Tapes 20mg original	14,324	2.27	—
46	Hemostatics	Tranexamic Acid 250mg original	13,866	2.20	—
47	Hyperlipidemia agents	Ezetimibe 10mg original	13,846	2.20	—
48	Acting mainly on gram-positive bacteria and mycoplasma	Clarithromycin 200mg original	13,319	2.11	—
49	Antidiarrheals, intestinal regulators	Antibiotics-Resistant Lactic Acid Bacteriae original	13,291	2.11	—
50	Other allergic agents	Desloratadine 5mg original	12,688	2.01	—

The 50 most-prescribed medications are described according to their therapeutic category, nonproprietary names, whether they are original or generic products, and their dosage.

Therapeutic categories were allocated according to the package insert or the Prescription Medications in Pharmaceuticals and Medical Devices Agency's search system [20].
