# Supplementary material for: Comorbidities and Concomitant Medications in Middle-Aged Japanese People According to the Charlson Comorbidity Index and Age: Results of the NDB-K7Ps-Study-3
Source: Epidemiologia (Basel). 2026 Mar 2;7(2):34. doi: 10.3390/epidemiologia7020034 (PMC13010749; doi:10.3390/epidemiologia7020034)
Supplement: Supplementary file 1 [file epidemiologia-07-00034-s001.zip › Table S2.pdf]

Table S2. Definitions of Charlson Comorbidity Index

Points	Charlson comorbidities	Corresponding ICD-10 codes	Number of diagnoses[*1]	Other criteria in our study	Unknown definition in our data[*2]
1	Myocardial infarction	I210-219, I220, I221, I228, I229, I252	52	—	with Hospitalization and with ECG change and/or cardiac marker elevation
	Congestive heart failure	I099, I110, I255, I420, I425-429, I430 (A188), I431 (E888), I431/E889, I432 (E639), I438 (E059), I500-509	44	—	NYHA functional classification II to IV symptoms
	Peripheral vascular disease	I700-702, I708-716, I718, I731, I738, I739, I771, K551, K552, K558, K559	101	—	with intermittent claudication or past bypass for chronic arterial insufficiency, history of gangrene or acute arterial insufficiency, or untreated thoracic or abdominal aneurysm (≥ 6 cm)
	Dementia	B220, E756, F010-012, F019, F03, F051, F107, G10, G20, G238, G300, G301, G308-311, G318	28	—	with chronic cognitive impairment
	Cerebrovascular disease (including TIA)	G450, G451, G453, G454, G458, G459, H340, I600-611, I613-616, I618-621, I629-636, I638, I639, I64, I650-653, I660-663, I668-679, I690, I691, I693, I694	248	—	with no or minor sequelae
	Chronic pulmonary disease (including asthma)	I278, I279, J40-47, J60, J61, J628, J630-635, J64, J65, J660, J661, J670-679, J684, J701, J703	114	—	—
	Rheumatic disease (Lupus, polymyositis, mixed connective tissue disease, polymyalgia rheumatica, moderate to severe rheumatoid arthritis)	M0500, M0510, M0520, M0530, M0580-0588, M0590-0598, M0600-0608, M0610, M0620, M0630, M0640, M0680-0688, M0690-0698, M320, 321, 329-332, 339-341, M348-M351, M353	135	—	—
	Peptic ulcer disease	K250-K257, K259-267, K269, K270, K279, K284,	60	—	Any history of treatment for ulcer

	(including ulcer bleeding)	K285, K287, K289			disease
2	Hemiplegia/ paraplegia	G114, G801, G802, G810, G811, G819, G820-825, G830-834, G839	43	—	—
	Moderate or severe renal disease (including uremia)	I120, N032-034, N036, N037, N052-057, N183-185, N189, N19, N250	42	—	Serum creatinine > 3.0mg/dL, dialysis or status post kidney transplant
6	AIDS<br>(including HIV infection)	B24, B200, B202-206, B210-212, B220, B221, B238	17	—	—
1	Mild liver disease<br>(Chronic hepatitis or cirrhosis without portal hypertension)	B181, B182, B189, K700-703, K709, K713, K717, K730, 732, 738-741, K743-746, K760, K762-764, K768, K769	86	—	—
3	Moderate and severe liver disease<br>(Chronic hepatitis or cirrhosis with portal hypertension with/without variceal bleeding)	I850, I859, I864, K704, K711, K721, K729, K765, K766, K767	35	—	—
1	Diabetes without chronic complication<br>(Diabetes with medication)	E100, E101, E106, E109, E11, E119, E12, E13, E139, E14, E149, E831, E881, E888, E891	30	including patients who were prescribed 487 oral hypoglycemic agent or 61 insulin preparation without insulin for intravenous injection (vial formulation)	—
2	Diabetes with chronic complication<br>—Retinopathy, neuropathy, or nephropathy	E102-105, E112-115, E132-135, E143-145, E888	41	including patients who were prescribed drug for diabetes neuropathy (mexiletine hydrochloride)	—
	— Hospitalized DKA or HHS	E100, E106, E116, E101, E111, E131, E140, E141,	38	including patients who prescribed	

		E110, E130		insulin for intravenous injection (vial formulation)	
2	Tumor/ lymphoma/ leukemia	C000-004, C006, C008, C009, C01, C020-022, C029-031, C039-041, C049-052, C059-062, C069-07, C080-081, C089-091, C100-104, C109-113, C119, C12, C131,C132, C139, C140, C150-155, C158-166, C169-172, C179-187, C189, C19, C20, C210-211, C220-224, C227, C229, C23, C240, C241, C248-254, C257-259, C261, C269-301, C310-313, C319-323, C329, C33, C340-343, C348-349, C37, C380-384, C400-403, C410-414, C419, C430-447, C449-452, C459, C469-476, C479-482, C490-496, C499, C500-506, C508-512, C519, C52, C530, C531, C538-543, C549, C55, C56, C570, C579, C58, C600-602, C609, C61, C620, C621, C629-632, C637, C639, C64, C65, C66, C670-677, C679, C681, C690-696, C700, C701, C709, C710-721,C723-725, C729, C73, C740, C741, C749-755, C760-765, C795, C809-814, C817, C819-821, C823, C824, C826, C827, C829-831, C833, C835, C837, C838, C840, C841, C844-848, C851, C852, C859-863, C865, C866, C880, C882-884, C900-903, C910, C911, C913-925, C927-931, C933, C939, C940, C942-944, C946, C947, C950, C951, C959, C960, C962, C964-966, C968,<br>D000-002, D011-014, D019-023, D043-047, D049-051, D059-061, D069-073, D090, D092,	1738	—	—

		D099, D471, D475, D648, D728, D763, H350, L270			
6	Metastatic tumor	C770-775, C778, C779, C780-788, C790-799, C800, C809	158	including patients with end-stage cancer and unknown primary cancer	

AIDS, acquired immunodeficiency syndrome; DKA, diabetic ketoacidosis; ECG, electrocardiogram; HHS, hyperosmolar hyperglycemic syndrome; HIV, human immunodeficiency virus; TIA, transient ischemic attack; ICD-10, International Classification of Diseases, 10th Revision; NYHA, New York Heart Association.

*1 Detail number of Japanese diagnostic code.

*2 Definitions according to the original Charlson Comorbidity Index [1].
