# Supplementary material for: Comorbidities and Concomitant Medications in Middle-Aged Japanese People According to the Charlson Comorbidity Index and Age: Results of the NDB-K7Ps-Study-3
Source: Epidemiologia (Basel). 2026 Mar 2;7(2):34. doi: 10.3390/epidemiologia7020034 (PMC13010749; doi:10.3390/epidemiologia7020034)
Supplement: Supplementary file 1 [file epidemiologia-07-00034-s001.zip › Table S21.pdf]

Table S21. Proportion of individuals diagnosed among those prescribed specific medications

Hypertension		Prescribed a specific medication	
n (% column)		yes	no
Diagnosis	yes	1,709,505 (72.1)	660,116 (27.9)
	SBP (mmHg)	133.0 ± 16.6	133.2 ± 17.1
	DBP (mmHg)	80.4 ± 11.6	80.7 ± 11.8
	no	60,998 (0.78)	7,753,000 (99.2)
	SBP (mmHg)	124.5 ± 17.1	120.6 ± 16.7
	DBP (mmHg)	75.8 ± 11.7	74.6 ± 11.7
		1,770,503 (17.4)	8,413,116 (82.6)

Sensitivity: 0.721 (0.721-0.722), Specificity: 0.992(0.992-0.992),
PPV: 0.966 (0.965-0.966), NPV: 0.922 (0.921-0.922), κ : 0.783 (0.782-0.783)

Diabetes		Prescribed a specific medication	
n (% column)		yes	no
Diagnosis	yes	535,545 (41.7)	750,145 (58.4)
	HbA_{1c} (%)	7.0 ± 1.2	6.0 ± 0.8
	no	424,400 (4.77)	8,473,529 (95.2)
	HbA_{1c} (%)	5.5 ± 0.4	5.5 ± 0.4
		959,945 (9.43)	9,223,674 (90.6)

Sensitivity: 0.417 (0.416-0.417), Specificity: 0.952 (0.952-0.952), PPV: 0.558 (0.557-0.559), NPV: 0.919 (0.919-0.919), κ : 0.414 (0.413-0.415)

Dyslipidemia		Prescribed a specific medication	
n (% column)		yes	no
Diagnosis	yes	1,308,082 (56.0)	1,027,563 (44.0)
	LDL (mg/dL)	119.4 ± 33.8	130.8 ± 33.0
	TG (mg/dL)	111 (80-159)	108 (76-158)
	no	54,373 (0.69)	7,793,601 (99.3)
	LDL (mg/dL)	122.4 ± 32.0	125.0 ± 30.6
	TG (mg/dL)	95 (66-140)	85 (60-126)
		1,362,455 (13.4)	8,821,164 (86.6)

Sensitivity: 0.560 (0.560-0.561), Specificity: 0.993 (0.993-0.993), PPV: 0.960 (0.960-0.960), NPV: 0.884 (0.883-0.884), κ : 0.648 (0.647-0.649)

Our data included 2000 antihypertensive agents and 487 oral hypoglycemic agents or 61 insulin preparations. We excluded insulin for intravenous injection (vial formulation). Our data included 531 medications for dyslipidemia, of which 391 were statins.

DBP, diastolic blood pressure; HbA1c, glycated hemoglobin; κ , kappa coefficient; LDL, low-density lipoprotein cholesterol; NPV, Negative predictive value; SBP, systolic blood pressure; TG, triglycerides; PPV, Positive predictive value.
