# Supplementary material for: Comorbidities and Concomitant Medications in Middle-Aged Japanese People According to the Charlson Comorbidity Index and Age: Results of the NDB-K7Ps-Study-3
Source: Epidemiologia (Basel). 2026 Mar 2;7(2):34. doi: 10.3390/epidemiologia7020034 (PMC13010749; doi:10.3390/epidemiologia7020034)
Supplement: Supplementary file 1 [file epidemiologia-07-00034-s001.zip › Table S22.pdf]

Table S22. Associations of CCI ≥ 4 in age 40-44 group with diagnosed diseases compared with CCI = 0 in age 70-74 group.

Diagnosed diseases	OR (95%CI)
Type 2 diabetes mellitus	7.00 (6.40-7.65)
Hypertension	4.31 (3.99-4.65)
Hyperlipidemia	3.51 (3.43-3.59)
Iron deficiency anemia	23.9 (22.1-25.8)
Hypothyroidism	11.3 (10.1-12.7)
Intractable reflux esophagitis	6.41 (5.82-7.07)
Gastritis	2.10 (1.96-2.26)
Constipation	3.84 (3.61-4.09)
Diarrhea	7.38 (6.76-8.06)
Depressive episodes	5.87 (5.32-6.47)
Sleep disorders	2.84 (2.67-3.03)
Anxiety neurosis	3.47 (3.11-3.87)

Data show Odds ratios (ORs) and 95% confidence intervals (CIs). The models were adjusted for potential confounders: sex, BMI, SBP, DBP, TG, LDL-C, HDL-C, HbA1c, pharmacotherapy for hypertension, diabetes, and dyslipidemia, smoking status, habitual exercise (≥30 minutes per session, >2 times/week vs. less frequent), and habitual alcohol consumption and frequency. All associations were statistically significant ($p < 0.0001$)
