# Supplementary material for: Comorbidities and Concomitant Medications in Middle-Aged Japanese People According to the Charlson Comorbidity Index and Age: Results of the NDB-K7Ps-Study-3
Source: Epidemiologia (Basel). 2026 Mar 2;7(2):34. doi: 10.3390/epidemiologia7020034 (PMC13010749; doi:10.3390/epidemiologia7020034)
Supplement: Supplementary file 1 [file epidemiologia-07-00034-s001.zip › Table S23.pdf]

Table S23. Consistency of diagnosed diseases between 2016 and 2018 (sensitivity, specificity, and kappa coefficient)

Hypertension		Diagnosis 2016		
n (% column)		yes	no	Totals
Diagnosis 2018	yes	1,539,930 (92.9)	117,449 (7.09)	1,287,355
	no	110,514 (8.58)	1,176,841 (91.4)	1,657,379
	Totals	1,650,444	1,294,290	2,944,734

Sensitivity: 0.933 (0.933-0.933), Specificity: 0.909 (0.909-0.910),
PPV: 0.929 (0.929-0.930), NPV: 0.914 (0.914-0.915), κ: 0.843 (0.842-0.843)

Diabetes *1		Diagnosis 2016		
n (% column)		yes	no	Totals
Diagnosis 2018	yes	747,601 (83.3)	150,221 (16.7)	2,046,912
	no	157,196 (7.68)	1,889,716 (92.3)	897,822
	Totals	2,039,937	904,797	2,944,734

Sensitivity: 0.826 (0.826-0.827), Specificity: 0.926 (0.926-0.927),
PPV: 0.833 (0.832-0.833), NPV: 0.923 (0.923-0.924), κ: 0.754 (0.753-0.755)

Dyslipidemia*2		Diagnosis 2016		
n (% column)		yes	no	Totals
Diagnosis 2018	yes	1,412,923 (89.7)	162,336 (10.3)	1,369,475
	no	210,592 (15.4)	1,158,883 (84.6)	1,575,259
	Totals	1,321,219	1,623,515	2,944,734

Sensitivity: 0.870 (0.870-0.871), Specificity: 0.877 (0.877-0.878),
PPV: 0.897 (0.896-0.897), NPV: 0.846 (0.846-0.847), κ : 0.745 (0.744-0.746)

Note: *1—all diabetes including type 1 diabetes;
*2—dyslipidemia including familial hyperlipidemia.
κ , kappa coefficient; NPV, Negative predictive value; PPV, Positive predictive value.
