# Supplementary material for: Comorbidities and Concomitant Medications in Middle-Aged Japanese People According to the Charlson Comorbidity Index and Age: Results of the NDB-K7Ps-Study-3
Source: Epidemiologia (Basel). 2026 Mar 2;7(2):34. doi: 10.3390/epidemiologia7020034 (PMC13010749; doi:10.3390/epidemiologia7020034)
Supplement: Supplementary file 1 [file epidemiologia-07-00034-s001.zip › Table S3,4.pdf]

Table S3. Prevalence of diagnosed diseases in all included individuals

Order	Names of Diagnoses	Corresponding ICD-10 Code	N	%
1	Allergic rhinitis	J304	3,065,920	30.1
2	Hypertension	I10	2,273,580	22.3
3	Acute bronchitis	J209	1,720,087	16.9
4	Allergic conjunctivitis	H101	1,663,753	16.3
5	Astigmatism	H522	1,660,681	16.3
6	Acute upper respiratory infection	J069	1,379,966	13.6
7	Chronic gastritis	K295	1,308,946	12.9
8	Hyperlipidemia	E785	1,105,219	10.9
9	Asthmatic bronchitis	J459	1,022,058	10.0
10	Low back pain	M5456	1,013,491	10.0
11	Pure hypercholesterolemia	E780	994,931	9.77
12	Reflux esophagitis	K210	962,915	9.46
13	Gastritis	K297	959,187	9.42
14	Unspecified diabetes mellitus	E14	932,525	9.16
15	Acute laryngopharyngitis	J060	906,869	8.91
16	Sleep disorders	G470	868,176	8.53
17	Eczema	L309	790,078	7.76
18	Constipation	K590	775,587	7.62
19	Acute pharyngitis	J029	672,645	6.61
20	Dyslipidemia	E785	670,287	6.58
21	Gastric ulcer	K259	662,972	6.51
22	Pharyngitis	J029	613,103	6.02
23	Hyperuricemia	E790	610,144	5.99
24	Acute sinusitis	J019	597,165	5.86
25	Dry eye syndrome	H041	585,108	5.75
26	Cataract	H269	579,856	5.69
27	Gonarthrosis	M171	558,320	5.48
28	Disorder of peripheral nervous system	G629	512,869	5.04
29	Hypermetropic astigmatism	H522	509,477	5.00
30	Acute gastritis	K291	480,256	4.72
31	influenza A	J101	460,008	4.52
32	Osteoporosis	M8199	458,851	4.51
33	Presbyopia	H524	457,995	4.50
34	Conjunctivitis	H109	446,724	4.39
35	Bronchitis	J40	440,901	4.33
36	Intractable reflux esophagitis with maintenance therapy	K210	422,969	4.15
37	Asteatosis	L853	419,372	4.12

38	Type 2 diabetes mellitus	E11	415,373	4.08
39	Asteatotic eczema	L853	402,859	3.96
40	Liver dysfunction	K769	396,117	3.89
41	Fatty liver	K760	383,309	3.76
42	Iron deficiency anemia	D509	375,386	3.69
43	Common cold	J00	360,860	3.54
44	Glaucoma	H409	341,732	3.36
45	Angina pectoris	I209	321,376	3.16
46	Chronic sinusitis	J329	320,668	3.15
47	Diarrhea	A099	312,345	3.07
48	Polyp of colon	K635	311,063	3.05
49	Keratoconjunctivitis sicca	H168	307,571	3.02
50	Asthenopia	H531	306,668	3.01
51	Atrophic gastritis	K294	293,878	2.89
52	Hyperplasia of prostate	N40	291,846	2.87
53	Urticaria	L509	287,392	2.82
54	Lumbar spondylosis deformans	M4786	285,973	2.81
55	Tinea pedis	B353	278,219	2.73
56	Leiomyoma of uterus	D259	274,837	2.70
57	Atopic dermatitis	L209	271,142	2.66
58	Depressive episode	F329	270,829	2.66
59	Lumbar spinal stenosis	M4806	261,076	2.56
60	Local infection of skin	L089	260,928	2.56
61	Enlarged optic nerve head excavation	H400	258,051	2.53
62	Anxiety neurosis	F411	253,256	2.49
63	Dehydration	E86	246,187	2.42
64	Dermatitis	L309	234,383	2.30
65	Contact dermatitis	L259	231,177	2.27
66	Tonsillitis	J039	228,352	2.24
67	Laryngopharyngitis	J060	225,357	2.21
68	Influenza, virus not identified	J111	219,176	2.15
69	Helicobacter pylori infection	A498	210,656	2.07
70	Intractable reflux esophagitis	K210	206,231	2.03
71	Pollinosis	J301	202,656	1.99
72	Neurotic disorder	F489	202,113	1.98
73	Neuropathic pain	G98	200,720	1.97
74	Blepharitis	H010	200,463	1.97
75	Seborrheic dermatitis	L219	197,282	1.94
76	Myopia	H521	196,458	1.93
77	Acute dermatitis	L309	192,245	1.89

78	Gout	M1009	191,568	1.88
79	Chronic bronchitis	J42	189,889	1.86
80	Myalgia	M7919	188,391	1.85
81	Impacted cerumen	H612	184,622	1.81
82	lumbar disc disorder	M519	183,827	1.81
83	Acute tonsillitis	J039	179,994	1.77
84	Cystitis	N309	179,317	1.76
85	lumbar disc herniation	M512	178,453	1.75
86	Cardiac arrhythmia	I499	178,114	1.75
87	Cervical spondylosis	M4782	175,449	1.72
88	Menopausal disorder	N951	173,341	1.70
89	Polyp of stomach	K317	167,588	1.65
90	Cervical spondylosis deformans	M4782	163,920	1.61
91	Internal hemorrhoid	K649	162,016	1.59
92	Peripheral neuropathic pain	G64	157,243	1.54
93	Stomatitis	K121	157,200	1.54
94	Migraine	G439	155,603	1.53
95	Overactive bladder	N328	150,762	1.48
96	Sensorineural hearing loss	H905	148,916	1.46
97	Pruritus	L299	147,723	1.45
98	Anemia	D649	145,884	1.43
99	Irritable bowel syndrome	K589	144,978	1.42
100	Unspecified diabetes retinopathy	E143	144,555	1.42
101	Chronic heart failure	I509	137,414	1.35
102	Mixed astigmatism	H522	137,045	1.35
103	High myopia	H521	135,964	1.34
104	Sleep apnea	G473	134,190	1.32
105	Chronic dermatitis	L309	133,879	1.31
106	Acute laryngitis	J040	132,976	1.31
107	Heart failure	I509	132,068	1.30
108	Senile cataract	H259	130,581	1.28
109	Disorder of breast	N649	130,018	1.28
110	Arteriosclerosis obliterans	I709	127,557	1.25
111	Primary ovarian failure	E283	126,532	1.24
112	Essential hypertension	I10	125,897	1.24
113	Rheumatoid arthritis	M0690	122,156	1.20
114	Otitis externa	H609	120,184	1.18
115	Sinusitis	J329	119,815	1.18
116	Cerebral infarction	I639	118,158	1.16
117	Chronic hepatitis	K739	117,247	1.15

118	Osteoarthrosis	M1999	116,827	1.15
119	Calculus of gallbladder	K802	116,776	1.15
120	Hypothyroidism	E039	115,763	1.14
121	Meniere's disease	H810	115,639	1.14
122	Postinflammatory hyperpigmentation	L810	114,247	1.12
123	Corneal ulcer	H160	109,184	1.07
124	Hemorrhoids	K649	108,611	1.07
125	Tinea unguium	B351	107,818	1.06
126	Otitis externa eczematoid	H605	107,039	1.05
127	Renal disease	N289	105,629	1.04

The 100 most-diagnosed diseases (or those with >1% prevalence) in all included individuals are described.

ICD-10: International Classification of Diseases, 10th Revision.

Table S4. Prevalence of prescribed medications in all included individuals

Order	Therapeutic Category	Drug name	N	%	number of generic drugs
1	Antipyretics and analgesics, anti-inflammatory agents	Loxoprofen Sodium Hydrate 60mg generic	1,625,762	16.0	18
2	Peptic ulcer agents	Rebamipide 100mg generic	1,565,015	15.4	28
3	Expectorants	L-Carbocisteine 500mg generic	1,242,743	12.2	5
4	Hemostatics	Tranexamic Acid 250mg generic	832,427	8.17	3
5	Antipyretics and analgesics, anti-inflammatory agents	Acetaminophen 200mg original	826,003	8.11	—
6	Acting mainly on gram-positive bacteria and mycoplasma	Clarithromycin 200mg generic	629,535	6.18	14
7	Other allergic agents	Fexofenadine Hydrochloride 60mg generic	592,773	5.82	31
8	Antitussives	Dextromethorphan Hydrobromide Hydrate 15mg generic	577,288	5.67	2
9	Common cold drugs	Salicylamide/Acetaminophen/Anhydrous Caffeine/ Promethazine Methylenedisalicylate original	564,114	5.54	3
10	Other allergic agents	Montelukast Sodium 10mg generic	543,802	5.34	39
11	Acting mainly on gram-positive and gram-negative bacteria	Cefcapene Pivoxil Hydrochloride Hydrate 100mg generic	521,540	5.12	5
12	Expectorants	L-Carbocisteine 250mg generic	488,884	4.80	5
13	Ophthalmic agents	Olopatadine Hydrochloride Solution 0.1% original	453,098	4.45	—
14	Antipyretics and analgesics, anti-inflammatory agents	Loxoprofen Sodium Hydrate 60mg original	438,888	4.31	—
15	Other allergic agents	Bilastine 20mg original	419,665	4.12	—
16	Antitussives	Dihydrocodeine Phosphate/dl-Methylephedrine Hydrochloride/ Chlorpheniramine Maleate original	393,262	3.86	—
17	Other allergic agents	Levocetirizine Hydrochloride 5mg original	383,144	3.76	—
18	Other digestive organ agents	Dequalinium Chloride Troches 0.25mg generic	362,891	3.56	—
19	Antitussives and expectorants	Tipepidine Hibenzate 20mg original	345,287	3.39	—
20	Antipyretics and analgesics, anti-inflammatory agents	Acetaminophen 300mg original	343,955	3.38	—
21	Antipyretics and analgesics, anti-inflammatory agents	Celecoxib 100mg original	339,625	3.34	—
22	Hyperlipidemia agents	Rosuvastatin Calcium 2.5mg generic	323,649	3.18	40

23	Antitussives	Dextromethorphan Hydrobromide Hydrate 15mg original	318,049	3.12	—
24	Otic and nasal agents	Fluticasone Furoate 27.5μg metered Nasal Spray original	310,090	3.04	—
25	Antipyretics and analgesics, anti-inflammatory agents	Acetaminophen 500mg original	304,612	2.99	—
26	Other allergic agents	Bepotastine Besilate 10mg generic	303,605	2.98	13
27	Expectorants	L-Carbocisteine 500mg original	301,392	2.96	—
28	Anticoagulants	Heparinoid cream/ lotion/ spray/ gel 0.3% generic	277,929	2.73	26
29	Antidiarrheals, intestinal regulators	Clostridium butyricum original	272,769	2.68	—
30	Peptic ulcer agents	Esomeprazole Magnesium Hydrate 20mg original	252,680	2.48	—
31	Vitamin B preparations	Mecobalamin 500μg original	248,933	2.44	—
32	Synthetic antibacterials	Garenoxacin Mesilate Hydrate 200mg original	248,525	2.44	—
33	Peptic ulcer agents	Rebamipide 100mg original	247,941	2.43	—
34	Antivirals	Baloxavir Marboxil 20mg original	243,906	2.40	—
35	Analgesics, anti-itchings, astringents, anti-inflammatory agents	Betamethasone Valerate/Gentamicin Sulfate Ointment 0.12% original	225,201	2.21	—
36	Antidiarrheals, intestinal regulators	Antibiotics-Resistant Lactic Acid Bacteriae original	224,982	2.21	—
37	Hemostatics	Tranexamic Acid 500mg generic	223,295	2.19	1
38	Anticoagulants	Heparinoid Ointment 0.3% original	215,590	2.12	—
39	Hemostatics	Tranexamic Acid 250mg original	204,994	2.01	—
40	Analgesics, anti-itchings, astringents, anti-inflammatory agents	Betamethasone Butyrate Propionate Ointment 0.05% original	199,189	1.96	—
41	Other allergic agents	Desloratadine 5mg original	198,216	1.95	—
42	Ophthalmic agents	Diquafosol Sodium Solution 3% original	197,547	1.94	—
43	Acting mainly on gram-positive and gram-negative bacteria	Cefcapene Pivoxil Hydrochloride Hydrate 100mg original	190,924	1.87	—
44	Otic and nasal agents	Mometasone Furoate Hydrate Nasal 50μg 56 sprays original	186,265	1.83	—
45	Antitussives	Dimemorfan Phosphate 10mg original	173,344	1.70	—
46	Acting mainly on gram-positive bacteria and mycoplasma	Clarithromycin 200mg original	169,643	1.67	—
47	Analgesics, anti-itchings, astringents, anti-inflammatory agents	Loxoprofen Sodium Hydrate Tapes 100mg original	167,991	1.65	—
48	Analgesics, anti-itchings, astringents, anti-inflammatory agents	Ketoprofen Tapes 40mg original	163,698	1.61	—

49	Analgesics, anti-itchings, astringents, anti-inflammatory agents	Hydrocortisone Butyrate Ointment 0.1% original	159,739	1.57	—
50	Antidiarrheals, intestinal regulators	Enterococcus faecium/ Clostridium butyricum/Bacillus subtilis original	153,698	1.51	—
	⋮	⋮	⋮	⋮	
	Antidiabetic agents	Metformin Hydrochloride 250mg original	81,290	0.80	—
	Antihypertensives	Azilsartan 20mg original	68,157	0.67	—

The 50 most-prescribed medications are described according to their therapeutic category, nonproprietary names, whether they are original or generic products, and their dosage.

Therapeutic categories were allocated according to the package insert or the Prescription Medications in Pharmaceuticals and Medical Devices Agency’s search system [16].
