# Supplementary material for: Comorbidities and Concomitant Medications in Middle-Aged Japanese People According to the Charlson Comorbidity Index and Age: Results of the NDB-K7Ps-Study-3
Source: Epidemiologia (Basel). 2026 Mar 2;7(2):34. doi: 10.3390/epidemiologia7020034 (PMC13010749; doi:10.3390/epidemiologia7020034)
Supplement: Supplementary file 1 [file epidemiologia-07-00034-s001.zip › Table S5-8.pdf]

Table S5. Prevalence of diagnosed diseases in the CCI=0, 40-44 age group

Order	Names of Diagnoses	Corresponding ICD-10 Code	N	%
1	Allergic rhinitis	J304	272,164	23.4
2	Acute bronchitis	J209	164,332	14.2
3	Astigmatism	H522	162,935	14.0
4	Allergic conjunctivitis	H101	146,848	12.6
5	Acute upper respiratory infection	J069	140,756	12.1
6	Acute laryngopharyngitis	J060	106,299	9.15
7	Acute sinusitis	J019	77,377	6.66
8	Acute pharyngitis	J029	73,295	6.31
9	Gastritis	K297	66,416	5.72
10	Chronic gastritis	K295	61,911	5.33
11	influenza A	J101	60,075	5.17
12	Eczema	L309	59,120	5.09
13	Acute gastritis	K291	51,454	4.43
14	Pharyngitis	J029	50,970	4.39
15	Low back pain	M5456	48,970	4.22
16	Reflux esophagitis	K210	38,610	3.33
17	Sleep disorders	G470	36,507	3.14
18	Atopic dermatitis	L209	36,239	3.12
19	Asteatosis	L853	33,717	2.90
20	Asteatosis eczema	L853	33,384	2.88
21	Dry eye syndrome	H041	33,104	2.85
22	Hypertension	I10	33,087	2.85
23	Conjunctivitis	H109	32,106	2.77
24	Common cold	J00	30,733	2.65
25	Leiomyoma of uterus	D259	30,723	2.65
26	Iron deficiency anemia	D509	29,392	2.53
27	Constipation	K590	28,894	2.49
28	Influenza, virus not identified	J111	28,531	2.46
29	Diarrhea	A099	28,295	2.44
30	Chronic sinusitis	J329	27,659	2.38
31	Urticaria	L509	25,644	2.21
32	Primary ovarian failure	E283	25,591	2.20
33	Local infection of skin	L089	25,453	2.19
34	Myopia	H521	23,034	1.98
35	Depressive episode	F329	22,103	1.90
36	Disorder of peripheral nervous system	G629	22,089	1.90
37	Tonsillitis	J039	21,483	1.85

38	Acute tonsillitis	J039	21,456	1.85
39	Laryngopharyngitis	J060	20,727	1.79
40	Keratoconjunctivitis sicca	H168	17,960	1.55
41	Contact dermatitis	L259	17,558	1.51
42	Acute dermatitis	L309	17,371	1.50
43	Hyperlipidemia	E785	17,031	1.47
44	Dermatitis	L309	17,001	1.46
45	Seborrheic dermatitis	L219	16,974	1.46
46	Acne vulgaris	L700	16,809	1.45
47	Hyperuricemia	E790	16,601	1.43
48	Presbyopia	H524	16,431	1.42
49	Dehydration	E86	16,181	1.39
50	Enlarged optic nerve head excavation	H400	15,910	1.37
51	Tinea pedis	B353	15,871	1.37
52	Blepharitis	H010	15,135	1.30
53	Acute laryngitis	J040	15,121	1.30
54	High myopia	H521	15,015	1.29
55	Neurotic disorder	F489	14,935	1.29
56	Migraine	G439	14,370	1.24
57	lumbar disc disorder	M519	14,244	1.23
58	lumbar disc herniation	M512	14,165	1.22
59	Pollinosis	J301	14,152	1.22
60	Dyslipidemia	E785	13,979	1.20
61	Asthenopia	H531	13,912	1.20
62	Hypercholesterolemia	E780	13,302	1.15
63	Impacted cerumen	H612	13,071	1.13
64	Glaucoma	H409	12,747	1.10
65	Disorder of breast	N649	12,680	1.09
66	Ovarian cyst	D27	11,736	1.01

The 100 most-diagnosed diseases and conditions (or those with >1% prevalence) in the CCI=0, 40-44 age group are described.

ICD-10: International Classification of Diseases, 10th Revision.

Table S6. Prevalence of diagnosed diseases in the CCI=0, 70-74 age group

Order	Names of Diagnoses	Corresponding ICD-10 Code	N	%
1	Hypertension	I10	150,073	32.7
2	Allergic rhinitis	J304	99,031	21.6
3	Cataract	H269	72,065	15.7
4	Allergic conjunctivitis	H101	68,161	14.9
5	Astigmatism	H522	64,738	14.1
6	Hypermetropic astigmatism	H522	60,468	13.2
7	Chronic gastritis	K295	59,937	13.1
8	Hyperlipidemia	E785	59,789	13.0
9	Hypercholesterolemia	E780	59,655	13.0
10	Osteoporosis	M8199	53,528	11.7
11	Acute bronchitis	J209	52,818	11.5
12	Sleep disorders	G470	50,311	11.0
13	Gonarthrosis	M171	49,365	10.8
14	Low back pain	M5456	48,351	10.5
15	Acute upper respiratory infection	J069	43,928	9.58
16	Gastritis	K297	40,727	8.88
17	Reflux esophagitis	K210	40,289	8.79
18	Constipation	K590	38,816	8.47
19	Dry eye syndrome	H041	38,222	8.34
20	Eczema	L309	36,761	8.02
21	Dyslipidemia	E785	33,061	7.21
22	Unspecified diabetes mellitus	E14	32,706	7.13
23	Conjunctivitis	H109	29,533	6.44
24	Presbyopia	H524	27,741	6.05
25	Glaucoma	H409	25,718	5.61
26	Lumbar spondylosis deformans	M4786	24,741	5.40
27	Acute laryngopharyngitis	J060	24,457	5.33
28	Lumbar spinal stenosis	M4806	23,778	5.19
29	Hyperplasia of prostate	N40	23,515	5.13
30	Disorder of peripheral nervous system	G629	23,267	5.07
31	Asthenopia	H531	21,933	4.78
32	Keratoconjunctivitis sicca	H168	20,564	4.49
33	Acute pharyngitis	J029	19,825	4.32
34	Hyperuricemia	E790	19,811	4.32
35	Asteatotic eczema	L853	19,243	4.20
36	Senile cataract	H259	17,577	3.83
37	Atrophic gastritis	K294	17,247	3.76

38	Asteatosis	L853	16,958	3.70
39	Pharyngitis	J029	16,912	3.69
40	Tinea pedis	B353	16,290	3.55
41	Acute sinusitis	J019	15,615	3.41
42	Enlarged optic nerve head excavation	H400	15,608	3.40
43	Intractable reflux esophagitis with maintenance therapy	K210	15,361	3.35
44	Polyp of colon	K635	15,359	3.35
45	Acute gastritis	K291	14,669	3.20
46	Mixed astigmatism	H522	13,819	3.01
47	Angina pectoris	I209	13,747	3.00
48	Contact dermatitis	L259	12,436	2.71
49	Overactive bladder	N328	12,378	2.70
50	Blepharitis	H010	11,930	2.60
51	Anxiety neurosis	F411	11,815	2.58
52	Dermatitis	L309	11,810	2.58
53	Impacted cerumen	H612	11,761	2.57
54	Common cold	J00	11,588	2.53
55	Preretinal membrane	H353	10,957	2.39
56	Myalgia	M7919	10,674	2.33
57	Trichiasis	H020	10,420	2.27
58	Neuropathic pain	G98	10,370	2.26
59	Chronic sinusitis	J329	9,955	2.17
60	influenza A	J101	9,800	2.14
61	Cervical spondylosis deformans	M4782	9,774	2.13
62	Cystitis	N309	9,529	2.08
63	Acute dermatitis	L309	9,476	2.07
64	Urticaria	L509	9,398	2.05
65	Local infection of skin	L089	9,285	2.03
66	Helicobacter pylori infection	A498	9,217	2.01
67	Seborrheic dermatitis	L219	8,929	1.95
68	Essential hypertension	I10	8,864	1.93
69	Sensorineural hearing loss	H905	8,848	1.93
70	Macular degeneration	H353	8,526	1.86
71	Cervical spondylosis	M4782	8,416	1.84
72	Dehydration	E86	8,389	1.83
73	Cardiac arrhythmia	I499	8,365	1.82
74	Spondylosis deformans	M4799	8,136	1.77
75	Tinea unguium	B351	7,966	1.74
76	Peripheral neuropathic pain	G64	7,854	1.71
77	Pollinosis	J301	7,832	1.71
78	Diarrhea	A099	7,809	1.70

79	Coxarthrosis	M169	7,733	1.69
80	Polyp of stomach	K317	7,598	1.66
81	Depressive episode	F329	7,318	1.60
82	Internal hemorrhoid	K649	7,198	1.57
83	Gout	M1009	7,196	1.57
84	Type 2 diabetes mellitus	E11	7,182	1.57
85	lumbar disc disorder	M519	7,165	1.56
86	Posterior vitreous detachment	H438	7,114	1.55
87	Neurotic disorder	F489	7,082	1.54
88	Osteoarthrosis	M1999	7,053	1.54
89	Intractable reflux esophagitis	K210	6,886	1.50
90	Corneal ulcer	H160	6,808	1.48
91	Seborrheic keratosis	L82	6,795	1.48
92	Normal tension glaucoma	H401	6,751	1.47
93	Pruritus	L299	6,501	1.42
94	Stomatitis	K121	6,334	1.38
95	Sciatica	M5438	6,214	1.36
96	Laryngopharyngitis	J060	6,208	1.35
97	Chronic dermatitis	L309	6,199	1.35
98	Hypermetropia	H520	6,168	1.35
99	Atopic dermatitis	L209	6,103	1.33
100	Myopia	H521	5,741	1.25
101	Iron deficiency anemia	D509	5,678	1.24
102	Conjunctival hemorrhage	H113	5,626	1.23
103	lumbar disc herniation	M512	5,179	1.13
104	Superficial punctate keratitis	H161	5,178	1.13
105	Otitis externa	H609	5,142	1.12
106	Arteriosclerotic fundus	H350	5,114	1.12
107	Meniere's disease	H810	5,050	1.10
108	Diffuse superficial keratitis	H161	5,027	1.10
109	Hemorrhoids	K649	4,885	1.07
110	Astigmatism	H522	4,878	1.06
111	Ringworm	B359	4,828	1.05
112	Ocular hypertension	H400	4,699	1.02
113	Helicobacter pylori gastritis	K296	4,667	1.02
114	Vitreous opacity	H433	4,665	1.02
115	Spondylolisthesis	M4316	4,629	1.01
116	Tonsillitis	J039	4,600	1.00

The 100 most-diagnosed diseases and conditions (or those with >1% prevalence) in the CCI=0, 70-74 age group are described.

ICD-10: International Classification of Diseases, 10th Revision.

Table S7. Prevalence of diagnosed diseases in the CCI ≥4, 40-44 age group

Order	Names of Diagnoses	Corresponding ICD-10 Code	N	%
1	Allergic rhinitis	J304	5,471	52.5
2	Hypertension	I10	4,155	39.9
3	Asthmatic bronchitis	J459	3,598	34.5
4	Unspecified diabetes mellitus	E14	3,564	34.2
5	Gastric ulcer	K259	3,557	34.2
6	Acute bronchitis	J209	3,271	31.4
7	Astigmatism	H522	3,064	29.4
8	Chronic gastritis	K295	2,917	28.0
9	Low back pain	M5456	2,864	27.5
10	Constipation	K590	2,718	26.1
11	Acute upper respiratory infection	J069	2,682	25.8
12	Hyperlipidemia	E785	2,631	25.3
13	Allergic conjunctivitis	H101	2,592	24.9
14	Sleep disorders	G470	2,416	23.2
15	Reflux esophagitis	K210	2,285	21.9
16	Hyperuricemia	E790	2,247	21.6
17	Liver dysfunction	K769	2,142	20.6
18	Iron deficiency anemia	D509	2,111	20.3
19	Hypercholesterolemia	E780	2,080	20.0
20	Type 2 diabetes mellitus	E11	2,027	19.5
21	Fatty liver	K760	1,944	18.7
22	Gastritis	K297	1,767	17.0
23	Eczema	L309	1,766	17.0
24	Acute laryngopharyngitis	J060	1,733	16.6
25	Dyslipidemia	E785	1,679	16.1
26	Pharyngitis	J029	1,564	15.0
27	Intractable reflux esophagitis with maintenance therapy	K210	1,546	14.8
28	Bronchitis	J40	1,524	14.6
29	Acute pharyngitis	J029	1,293	12.4
30	Asteatosis	L853	1,226	11.8
31	Disorder of peripheral nervous system	G629	1,191	11.4
32	Acute sinusitis	J019	1,152	11.1
33	Diarrhea	A099	1,151	11.1
34	Unspecified diabetes retinopathy	E143	1,143	11.0
35	Osteoporosis	M8199	1,114	10.7
36	Unspecified diabetic nephropathy	E142	1,077	10.3
37	Heart failure	I509	989	9.5

38	Chronic bronchitis	J42	978	9.4
39	Angina pectoris	I209	972	9.3
40	Intractable reflux esophagitis	K210	969	9.3
41	Chronic heart failure	I509	952	9.1
42	Chronic kidney failure	N189	951	9.1
43	Dry eye syndrome	H041	927	8.9
44	Common cold	J00	907	8.7
45	Asteatotic eczema	L853	904	8.7
46	Acute gastritis	K291	887	8.5
47	Depressive episode	F329	870	8.4
48	Malignant neoplasm of breast	C509	866	8.3
49	Chronic hepatitis	K739	845	8.1
50	Arteriosclerosis obliterans	I709	827	7.9
51	Rheumatoid arthritis	M0690	814	7.8
52	influenza A	J101	802	7.7
53	Congestive heart failure	I500	795	7.6
54	Chronic sinusitis	J329	787	7.6
55	Hypothyroidism	E039	769	7.4
56	Urticaria	L509	747	7.2
57	Atopic dermatitis	L209	746	7.2
58	Dehydration	E86	742	7.1
59	Local infection of skin	L089	734	7.0
60	Stomatitis	K121	696	6.7
61	Anxiety neurosis	F411	684	6.6
62	Conjunctivitis	H109	674	6.5
63	Anemia	D649	637	6.1
64	Tonsillitis	J039	622	6.0
65	Leiomyoma of uterus	D259	619	5.9
66	Renal disease	N289	612	5.9
67	Chronic kidney disease	N189	586	5.6
68	Neurotic disorder	F489	580	5.6
69	Primary ovarian failure	E283	562	5.4
70	Cardiac arrhythmia	I499	556	5.3
71	Pruritus	L299	543	5.2
72	Migraine	G439	521	5.0
73	Systemic lupus erythematosus	M329	520	5.0
74	Liver disease	K769	512	4.9
75	Dermatitis	L309	506	4.9
76	Renal anemia	N19	498	4.8
77	Atherosclerosis	I709	487	4.7
78	Irritable bowel syndrome	K589	473	4.5

79	Schizophrenia	F209	461	4.4
80	Tinea pedis	B353	456	4.4
81	Sleep apnea	G473	448	4.3
82	Laryngopharyngitis	J060	435	4.2
83	Peripheral neuropathic pain	G64	434	4.2
84	Keratoconjunctivitis sicca	H168	433	4.2
85	Gout	M1009	431	4.1
86	Polyp of colon	K635	428	4.1
87	Acute tonsillitis	J039	427	4.1
88	Neuropathic pain	G98	420	4.0
89	Pollinosis	J301	410	3.9
90	Influenza, virus not identified	J111	409	3.9
91	Peripheral vascular disease	I739	395	3.8
92	Cystitis	N309	388	3.7
93	Cervical lymph node metastasis	C770	388	3.7
94	Glaucoma	H409	387	3.7
95	Myalgia	M7919	386	3.7
96	Hemorrhoids	K649	384	3.7
97	Carotid atherosclerosis	I652	381	3.7
98	Thyroid cancer	C73	374	3.6
99	Contact dermatitis	L259	373	3.6
100	Asthenopia	H531	366	3.5

The 100 most-diagnosed diseases and conditions (or those with >1% prevalence) in the CCI ≥4, 40-44 age group are described.

ICD-10: International Classification of Diseases, 10th Revision.

Table S8. Prevalence of diagnosed diseases in the CCI ≥4, 70-74 age group

Order	Names of Diagnoses	Corresponding ICD-10 Code	N	%
1	Hypertension	I10	75,155	72.2
2	Unspecified diabetes mellitus	E14	50,906	48.9
3	Allergic rhinitis	J304	45,100	43.3
4	Hyperlipidemia	E785	42,306	40.6
5	Gastric ulcer	K259	41,390	39.8
6	Chronic gastritis	K295	38,749	37.2
7	Constipation	K590	38,507	37.0
8	Hypercholesterolemia	E780	35,818	34.4
9	Low back pain	M5456	32,468	31.2
10	Reflux esophagitis	K210	32,060	30.8
11	Sleep disorders	G470	31,490	30.2
12	Cataract	H269	27,519	26.4
13	Asthmatic bronchitis	J459	26,650	25.6
14	Astigmatism	H522	26,590	25.5
15	Type 2 diabetes mellitus	E11	25,688	24.7
16	Allergic conjunctivitis	H101	25,030	24.0
17	Angina pectoris	I209	24,427	23.5
18	Acute bronchitis	J209	24,399	23.4
19	Osteoporosis	M8199	24,310	23.3
20	Intractable reflux esophagitis with maintenance therapy	K210	23,889	22.9
21	Hypermetropic astigmatism	H522	23,229	22.3
22	Dyslipidemia	E785	21,834	21.0
23	Hyperuricemia	E790	21,227	20.4
24	Hyperplasia of prostate	N40	19,876	19.1
25	Gonarthrosis	M171	19,659	18.9
26	Gastritis	K297	19,308	18.5
27	Fatty liver	K760	19,300	18.5
28	Eczema	L309	18,664	17.9
29	Disorder of peripheral nervous system	G629	18,571	17.8
30	Acute upper respiratory infection	J069	18,374	17.6
31	Liver dysfunction	K769	18,248	17.5
32	Unspecified diabetes retinopathy	E143	16,194	15.6
33	Dry eye syndrome	H041	15,557	14.9
34	Polyp of colon	K635	14,938	14.3
35	Heart failure	I509	14,666	14.1
36	Iron deficiency anemia	D509	14,384	13.8
37	Lumbar spinal stenosis	M4806	14,324	13.8

38	Arteriosclerosis obliterans	I709	14,291	13.7
39	Chronic heart failure	I509	14,224	13.7
40	Cerebral infarction	I639	13,517	13.0
41	Intractable reflux esophagitis	K210	12,580	12.1
42	Chronic bronchitis	J42	12,278	11.8
43	Conjunctivitis	H109	12,175	11.7
44	Lumbar spondylosis deformans	M4786	12,077	11.6
45	Atrophic gastritis	K294	11,525	11.1
46	Acute laryngopharyngitis	J060	11,122	10.7
47	Bronchitis	J40	11,098	10.7
48	Asteatosis	L853	10,405	10.0
49	Presbyopia	H524	10,260	9.9
50	Asteatotic eczema	L853	10,260	9.9
51	Unspecified diabetic nephropathy	E142	10,108	9.7
52	Glaucoma	H409	10,104	9.7
53	Cardiac arrhythmia	I499	9,984	9.6
54	Pharyngitis	J029	9,950	9.6
55	Overactive bladder	N328	9,892	9.5
56	Malignant neoplasm of prostate	C61	9,577	9.2
57	Carotid artery stenosis	I652	9,202	8.8
58	Asthenopia	H531	9,040	8.7
59	Congestive heart failure	I500	8,630	8.3
60	Anxiety neurosis	F411	8,418	8.1
61	Keratoconjunctivitis sicca	H168	8,270	7.9
62	Tinea pedis	B353	8,174	7.9
63	Acute pharyngitis	J029	8,131	7.8
64	Carotid atherosclerosis	I652	8,018	7.7
65	Dehydration	E86	7,731	7.4
66	Rheumatoid arthritis	M0690	7,615	7.3
67	Chronic hepatitis	K739	7,253	7.0
68	Anemia	D649	7,229	6.9
69	Helicobacter pylori infection	A498	7,077	6.8
70	Atherosclerosis	I709	6,997	6.7
71	Diarrhea	A099	6,928	6.7
72	Senile cataract	H259	6,905	6.6
73	Atrial fibrillation	I489	6,752	6.5
74	Neuropathic pain	G98	6,747	6.5
75	Peripheral neuropathic pain	G64	6,681	6.4
76	Acute sinusitis	J019	6,671	6.4
77	Calculus of gallbladder	K802	6,614	6.4
78	Chronic sinusitis	J329	6,566	6.3

79	Common cold	J00	6,458	6.2
80	Cervical spondylosis deformans	M4782	6,221	6.0
81	Renal disease	N289	5,920	5.7
82	Myalgia	M7919	5,913	5.7
83	Mixed astigmatism	H522	5,910	5.7
84	Polyp of stomach	K317	5,819	5.6
85	Chronic kidney failure	N189	5,799	5.6
86	Hypothyroidism	E039	5,779	5.6
87	Dermatitis	L309	5,668	5.4
88	Depressive episode	F329	5,660	5.4
89	Malignant neoplasm of breast	C509	5,644	5.4
90	Cervical spondylosis	M4782	5,545	5.3
91	Gastric cancer	C169	5,524	5.3
92	Chronic kidney disease	N189	5,478	5.3
93	Stomatitis	K121	5,434	5.2
94	Cystitis	N309	5,287	5.1
95	Essential hypertension	I10	5,233	5.0
96	Old myocardial infarction	I252	5,140	4.9
97	Enlarged optic nerve head excavation	H400	5,105	4.9
98	Acute gastritis	K291	5,083	4.9
99	Cyst of kidney	N281	5,075	4.9
100	Internal hemorrhoid	K649	5,068	4.9

The 100 most-diagnosed diseases and conditions (or those with >1% prevalence) in the CCI ≥4, 70-74 age group are described.

ICD-10: International Classification of Diseases, 10th Revision.
