# Supplementary material for: Comorbidities and Concomitant Medications in Middle-Aged Japanese People According to the Charlson Comorbidity Index and Age: Results of the NDB-K7Ps-Study-3
Source: Epidemiologia (Basel). 2026 Mar 2;7(2):34. doi: 10.3390/epidemiologia7020034 (PMC13010749; doi:10.3390/epidemiologia7020034)
Supplement: Supplementary file 1 [file epidemiologia-07-00034-s001.zip › Table S9-12.pdf]

Table S9. Prevalence of prescribed medications in the CCI=0, 40-44 age group

Order	Therapeutic Category	Drug name	N	%	number of generic drugs
1	Antipyretics and analgesics, anti-inflammatory agents	Loxoprofen Sodium Hydrate 60mg generic	167,937	14.5	16
2	Peptic ulcer agents	Rebamipide 100mg generic	136,023	11.7	26
3	Expectorants	L-Carbocisteine 500mg generic	129,329	11.1	5
4	Hemostatics	Tranexamic Acid 250mg generic	94,081	8.10	3
5	Antipyretics and analgesics, anti-inflammatory agents	Acetaminophen 200mg original	93,904	8.09	—
6	Acting mainly on gram-positive bacteria and mycoplasma	Clarithromycin 200mg generic	58,155	5.01	13
7	Other allergic agents	Fexofenadine Hydrochloride 60mg generic	57,177	4.92	24
8	Antitussives	Dextromethorphan Hydrobromide Hydrate generic	53,938	4.65	2
9	Acting mainly on gram-positive and gram-negative bacteria	Cefcapene Pivoxil Hydrochloride Hydrate 100mg generic	53,901	4.64	5
10	Common cold drugs	Salicylamide/Acetaminophen/Anhydrous Caffeine/ Promethazine Methylenedisalicylate original	48,128	4.14	3
11	Expectorants	L-Carbocisteine 250mg generic	48,103	4.14	4
12	Other allergic agents	Bilastine 20mg original	45,106	3.88	—
13	Acting mainly on gram-positive and gram-negative bacteria	Cefditoren Pivoxil 100mg generic	44,726	3.85	5
14	Ophthalmic agents	Olopatadine Hydrochloride Solution 0.1% original	40,048	3.45	—
15	Other allergic agents	Levocetirizine Hydrochloride 5mg original	39,337	3.39	—
16	Antipyretics and analgesics, anti-inflammatory agents	Loxoprofen Sodium Hydrate 60mg original	38,658	3.33	—
17	Antipyretics and analgesics, anti-inflammatory agents	Acetaminophen 300mg original	36,784	3.17	—
18	Antipyretics and analgesics, anti-inflammatory agents	Acetaminophen 500mg original	35,377	3.05	—
19	Otic and nasal agents	Fluticasone Furoate 27.5μg metered Nasal Spray original	34,322	2.96	—
20	Antitussives and expectorants	Tipepidine Hibenzate 20mg original	33,988	2.93	—
21	Other digestive organ agents	Dequalinium Chloride Troches 0.25mg generic	33,605	2.89	—
22		Montelukast Sodium 10mg generic	33,553	2.89	29

23	Other allergic agents	Bepotastine Besilate 10mg generic	32,419	2.79	11
24	Antitussives	Dihydrocodeine Phosphate/dl-Methylephedrine Hydrochloride/ Chlorpheniramine Maleate original	32,400	2.79	—
25	Antivirals	Baloxavir Marboxil 20mg original	32,280	2.78	—
26	Antidiarrheals, intestinal regulators	Clostridium butyricum original	28,308	2.44	—
27	Hemostatics	Tranexamic Acid 500mg generic	28,023	2.41	1
28	Expectorants	L-Carbocisteine 500mg original	26,753	2.30	—
29	Synthetic antibacterials	Garenoxacin Mesilate Hydrate 200mg original	26,159	2.25	—
30	Antidiarrheals, intestinal regulators	Antibiotics-Resistant Lactic Acid Bacteriae original	25,988	2.24	—
31	Anticoagulants	Heparinoid cream/ lotion/ spray/ gel 0.3% generic	25,934	2.23	14
32	Antitussives	Dextromethorphan Hydrobromide Hydrate 15mg original	25,536	2.20	—
33	Anticoagulants	Heparinoid Ointment 0.3% original	20,596	1.77	—
34	Acting mainly on gram-positive and gram-negative bacteria	Amoxicillin Hydrate 250mg generic	20,351	1.75	2
35	Antivirals	Laninamivir Octanoate Hydrate 20mg original	20,197	1.74	—
36	Analgesics, anti-itchings, astringents, anti-inflammatory agents	Betamethasone Butyrate Propionate Ointment 0.05% original	20,042	1.73	—
37	Hemostatics	Tranexamic Acid 250mg original	19,690	1.70	—
38	Other allergic agents	Desloratadine 5mg original	19,104	1.65	—
39	Peptic ulcer agents	Rebamipide 100mg original	18,581	1.60	—
40	Otic and nasal agents	Mometasone Furoate Hydrate Nasal 50μg 56 sprays original	18,555	1.60	—
41	Analgesics, anti-itchings, astringents, anti-inflammatory agents	Betamethasone Valerate/Gentamicin Sulfate Ointment 0.12% original	17,790	1.53	—
42	Acting mainly on gram-positive and gram-negative bacteria	Cefcapene Pivoxil Hydrochloride Hydrate 100mg original	16,953	1.46	—
43	Analgesics, anti-itchings, astringents, anti-inflammatory agents	Hydrocortisone Butyrate Ointment 0.1% original	16,682	1.44	—
44	Antipyretics and analgesics, anti-inflammatory agents	Celecoxib 100mg original	16,318	1.41	—
45	Antidiarrheals, intestinal regulators	Enterococcus faecium/ Clostridium butyricum/Bacillus subtilis original	15,388	1.33	—
46	Antitussives	Dimemorfan Phosphate 10mg original	14,496	1.25	—
47	Other allergic agents	Rupatadine Fumarate 10mg original	14,342	1.24	—
48	Antidiarrheals, intestinal regulators	Bifidobacterium original	14,046	1.21	—

49	Acting mainly on gram-positive and gram-negative bacteria	Cefditoren Pivoxil 100mg original	13,997	1.21	—
50	Acting mainly on gram-positive bacteria and mycoplasma	Clarithromycin 200mg original	13,979	1.20	—

The 50 most-prescribed medications are described according to their therapeutic categories, nonproprietary names, whether they are original or generic products, and their dosage.

Therapeutic categories were allocated according to the package insert or the Prescription Medications in Pharmaceuticals and Medical Devices Agency's search system [20].

Table S10. Prevalence of prescribed medications in the CCI=0, 70-74 age group

Order	Therapeutic Category	Drug name	N	%	number of generic drugs
1	Peptic ulcer agents	Rebamipide 100mg generic	57,911	12.6	27
2	Antipyretics and analgesics, anti-inflammatory agents	Loxoprofen Sodium Hydrate 60mg generic	50,465	11.0	16
3	Expectorants	L-Carbocisteine 500mg generic	29,856	6.51	5
4	Analgesics, anti-itchings, astringents, anti-inflammatory agents	Loxoprofen Sodium Hydrate Tape/ Patch 100mg generic	29,591	6.45	26
5	Antipyretics and analgesics, anti-inflammatory agents	Acetaminophen 200mg original	22,217	4.85	—
6	Antipyretics and analgesics, anti-inflammatory agents	Celecoxib 100mg original	21,269	4.64	—
7	Hemostatics	Tranexamic Acid 250mg generic	20,558	4.48	3
8	Common cold drugs	Salicylamide/Acetaminophen/Anhydrous Caffeine/ Promethazine Methylenedisalicylate original	18,862	4.11	3
9	Acting mainly on gram-positive and gram-negative bacteria	Cefcapene Pivoxil Hydrochloride Hydrate 100mg generic	18,316	3.99	5
10	Hyperlipidemia agents	Rosuvastatin Calcium 2.5mg generic	18,241	3.98	26
11	Other allergic agents	Fexofenadine Hydrochloride 60mg generic	17,850	3.89	24
12	Ophthalmic agents	Olopatadine Hydrochloride Solution 0.1% original	17,764	3.87	—
13	Vitamins A and D preparations	Eldecalcitol 0.75μg original	16,437	3.59	—
14	Antitussives	Dextromethorphan Hydrobromide Hydrate 15mg generic	15,710	3.43	2
15	Expectorants	L-Carbocisteine 250mg generic	14,573	3.18	4
16	Antipyretics and analgesics, anti-inflammatory agents	Loxoprofen Sodium Hydrate 60mg original	14,506	3.16	—
17	Ophthalmic agents	Diquafosol Sodium Solution 3% original	12,164	2.65	—
18	Analgesics, anti-itchings, astringents, anti-inflammatory agents	Ketoprofen Tapes 40mg original	11,865	2.59	—
19	Vitamin B preparations	Mecobalamin 500μg original	11,560	2.52	—
20	Anticoagulants	Heparinoid cream/ lotion/ spray/ gel 0.3% generic	11,426	2.49	14
21	Other allergic agents	Bilastine 20mg original	11,089	2.42	—
22	Other digestive organ agents	Dequalinium Chloride 0.25mg generic	10,812	2.36	—
23	Analgesics, anti-itchings, astringents, anti-inflammatory agents	Betamethasone Valerate/Gentamicin Sulfate Ointment 0.12% original	10,607	2.31	—

24	Other allergic agents	Levocetirizine Hydrochloride 5mg original	10,479	2.29	—
25	Analgesics, anti-itchings, astringents, anti-inflammatory agents	Loxoprofen Sodium Hydrate Tapes 100mg original	10,412	2.27	—
26	Antitussives	Dextromethorphan Hydrobromide Hydrate 15mg original	9,813	2.14	—
27	Peptic ulcer agents	Rebamipide 100mg original	9,491	2.07	—
28	Antipyretics and analgesics, anti-inflammatory agents	Acetaminophen 300mg original	9,362	2.04	—
29	Antitussives and expectorants	Tipepidine Hibenzate 20mg original	9,247	2.02	—
30	Anticoagulants	Heparinoid Ointment 0.3% original	8,859	1.93	—
31	Peptic ulcer agents	Esomeprazole Magnesium Hydrate 20mg original	8,759	1.91	—
32	Analgesics, anti-itchings, astringents, anti-inflammatory agents	Betamethasone Butyrate Propionate Ointment 0.05% original	8,658	1.89	—
33	Expectorants	L-Carbocisteine 500mg original	7,745	1.69	—
34	Acting mainly on gram-positive and gram-negative bacteria	Cefcapene Pivoxil Hydrochloride Hydrate 100mg original	7,287	1.59	—
35	Antitussives	Dihydrocodeine Phosphate/ dl-Methylephedrine Hydrochloride/Chlorpheniramine Maleate original	7,223	1.58	—
36	Antacids	Magnesium Oxide 330mg generic	7,216	1.57	4
37	Antipyretics and analgesics, anti-inflammatory agents	Acetaminophen 500mg original	7,007	1.53	—
38	Analgesics, anti-itchings, astringents, anti-inflammatory agents	Hydrocortisone Butyrate Ointment 0.1% original	6,923	1.51	—
39	Antidiarrheals, intestinal regulators	Clostridium butyricum original	6,870	1.50	—
40	Other agents affecting metabolism	Adenosine Triphosphate Disodium Hydrate 10% original	6,759	1.47	—
41	Otic and nasal agents	Fluticasone Furoate 27.5μg metered Nasal Spray original	6,732	1.47	—
42	Ophthalmic agents	Moxifloxacin Hydrochloride Solution 0.5% original	6,404	1.40	—
43	Other agents affecting central nervous system	Pregabalin 25mg original	6,315	1.38	—
44	Antidiarrheals, intestinal regulators	Antibiotics-Resistant Lactic Acid Bacteriae original	5,776	1.26	—
45	Otic and nasal agents	Mometasone Furoate Hydrate Nasal 50μg 56 sprays original	5,636	1.23	—

46	Analgesics, anti-itchings, astringents, anti-inflammatory agents	Esflurbiprofen/Mentha Oil Tapes original	5,570	1.21	—
47	Other allergic agents	Desloratadine 5mg original	5,535	1.21	—
48	Ophthalmic agents	Levofloxacin Hydrate Solution 1.5% original	5,531	1.21	—
49	Hemostatics	Tranexamic Acid 250mg original	5,487	1.20	—
50	Analgesics, anti-itchings, astringents, anti-inflammatory agents	Ketoprofen Tapes 20mg original	5,310	1.16	—

The 50 most-prescribed medications are described according to their therapeutic categories, nonproprietary names, whether they are original or generic products, and their dosage.

Therapeutic categories were allocated according to the package insert or the Prescription Medications in Pharmaceuticals and Medical Devices Agency's search system [20].

Table S11. Prevalence of prescribed medications in the CCI ≥4, 40-44 age group

Order	Therapeutic Category	Drug name	N	%	number of generic drugs
1	Antipyretics and analgesics, anti-inflammatory agents	Loxoprofen Sodium Hydrate 60mg generic	3,059	29.4	16
2	Peptic ulcer agents	Rebamipide 100mg generic	2,784	26.7	27
3	Expectorants	L-Carbocisteine 500mg generic	2,549	24.5	4
4	Antipyretics and analgesics, anti-inflammatory agents	Acetaminophen 200mg original	1,785	17.1	—
5	Hemostatics	Tranexamic Acid 250mg generic	1,679	16.1	2
6	Other allergic agents	Montelukast Sodium 10mg generic	1,450	13.9	29
7	Acting mainly on gram-positive bacteria and mycoplasma	Clarithromycin generic	1,310	12.6	13
8	Antitussives	Dextromethorphan Hydrobromide Hydrate 15mg generic	1,192	11.4	2
9	Common cold drugs	Salicylamide/Acetaminophen/Anhydrous Caffeine/ Promethazine Methylenedisalicylate original	1,173	11.26	3
10	Acting mainly on gram-positive and gram-negative bacteria	Cefcapene Pivoxil Hydrochloride Hydrate 100mg generic	1,002	9.62	5
11	Expectorants	L-Carbocisteine 250mg generic	943	9.05	4
12	Antipyretics and analgesics, anti-inflammatory agents	Loxoprofen Sodium Hydrate 60mg original	923	8.86	—
13	Antitussives	Dihydrocodeine Phosphate/dl-Methylephedrine Hydrochloride/ Chlorpheniramine Maleate original	909	8.73	—
14	Antipyretics and analgesics, anti-inflammatory agents	Acetaminophen 500mg original	888	8.53	—
15	Other allergic agents	Bilastine 20mg original	819	7.86	—
16	Antipyretics and analgesics, anti-inflammatory agents	Acetaminophen 300mg original	798	7.66	—
17	Other digestive organ agents	Dequalinium Chloride 0.25mg generic	775	7.44	—
18	Antidiarrheals, intestinal regulators	Clostridium butyricum original	772	7.41	—
19	Antitussives	Dextromethorphan Hydrobromide Hydrate 15mg original	770	7.39	—
20	Peptic ulcer agents	Esomeprazole Magnesium Hydrate 20mg original	770	7.39	—
21	Other allergic agents	Levocetirizine Hydrochloride 5mg original	769	7.38	—
22	Expectorants	L-Carbocisteine 500mg original	753	7.23	—

23	Otic and nasal agents	Fluticasone Furoate 27.5μg metered Nasal Spray original	748	7.18	—
24	Ophthalmic agents	Olopatadine Hydrochloride Solution 0.1% original	704	6.76	—
25	Antitussives and expectorants	Tipepidine Hibenzate 20mg original	690	6.63	—
26	Anticoagulants	Heparinoid Cream/ Lotion/ Spray/ Gel 0.3% generic	662	6.36	14
27	Hyperlipidemia agents	Rosuvastatin Calcium 2.5 mg generic	635	6.10	26
28	Adrenal hormone preparations	Prednisolone 5mg original	633	6.08	—
29	Synthetic antibacterials	Garenoxacin Mesilate Hydrate 200mg original	628	6.03	—
30	Gout preparations	Febuxostat 10mg original	597	5.73	—
31	Antidiarrheals, intestinal regulators	Antibiotics-Resistant Lactic Acid Bacteriae original	548	5.26	—
32	Anticoagulants	Heparinoid Ointment 0.3% original	525	5.04	—
33	Peptic ulcer agents	Rebamipide 100mg original	523	5.02	—
34	Hemostatics	Tranexamic Acid 500mg generic	517	4.96	1
35	Other blood and body fluid agents	Aspirin 100mg original	473	4.54	—
36	Hemostatics	Tranexamic Acid 250mg original	467	4.48	—
37	Antidiabetic agents	Metformin Hydrochloride 250mg original	464	4.46	—
38	Gout preparations	Febuxostat 20mg original	436	4.19	—
39	Vitamin B preparations	Mecobalamin 500μg original	431	4.14	—
40	Analgesics, anti-itchings, astringents, anti-inflammatory agents	Betamethasone Valerate/Gentamicin Sulfate Ointment 0.12% original	427	4.10	—
41	Antipyretics and analgesics, anti-inflammatory agents	Celecoxib 100mg original	421	4.04	—
42	Other respiratory organ agents	Budesonide/Formoterol Fumarate Hydrate Turbuhaler 60 doses original	411	3.95	—
43	Antidiarrheals, intestinal regulators	Bifidobacterium original	408	3.92	—
44	Peptic ulcer agents	Vonoprazan Fumarate 10mg original	401	3.85	—
45	Other allergic agents	Desloratadine 5mg original	394	3.78	—
46	Analgesics, anti-itchings, astringents, anti-inflammatory agents	Betamethasone Butyrate Propionate Ointment 0.05% original	389	3.74	—
47	Acting mainly on gram-positive and gram-negative bacteria	Cefcapene Pivoxil Hydrochloride Hydrate 100mg original	388	3.73	—
48	Acting mainly on gram-positive bacteria and mycoplasma	Clarithromycin 200mg original	385	3.70	—
49	Peptic ulcer agents	Vonoprazan Fumarate 20mg original	371	3.56	—
50	Antivirals	Baloxavir Marboxil 20mg original	370	3.55	—

The 50 most-prescribed medications are described according to their therapeutic categories, nonproprietary names, whether they are original or generic products, and their dosage.

Therapeutic categories were allocated according to the package insert or the Prescription Medications in Pharmaceuticals and Medical Devices Agency's search system [20].

Table S12. Prevalence of prescribed medications in the CCI ≥4, 70-74 age group

Order	Therapeutic Category	Drug name	N	%	number of generic drugs
1	Peptic ulcer agents	Rebamipide 100mg generic	22,923	22.0	26
2	Antipyretics and analgesics, anti-inflammatory agents	Loxoprofen Sodium Hydrate 60mg generic	19,471	18.7	16
3	Expectorants	L-Carbocisteine 500mg generic	15,925	15.3	5
4	Other blood and body fluid agents	Aspirin 100mg original	13,401	12.9	—
5	Antipyretics and analgesics, anti-inflammatory agents	Acetaminophen 200mg original	10,970	10.5	—
6	Hyperlipidemia agents	Rosuvastatin Calcium 2.5mg generic	9,777	9.39	26
7	Peptic ulcer agents	Esomeprazole Magnesium Hydrate 20mg original	9,270	8.90	—
8	Common cold drugs	Salicylamide/Acetaminophen/Anhydrous Caffeine/ Promethazine Methylenedisalicylate original	9,007	8.65	3
9	Hemostatics	Tranexamic Acid 250mg generic	8,505	8.17	3
10	Antipyretics and analgesics, anti-inflammatory agents	Celecoxib 100mg original	8,319	7.99	—
11	Antitussives	Dextromethorphan Hydrobromide Hydrate 15mg generic	7,964	7.65	2
12	Acting mainly on gram-positive and gram-negative bacteria	Cefcapene Pivoxil Hydrochloride Hydrate 100mg generic	7,599	7.30	5
13	Expectorants	L-Carbocisteine 250mg generic	7,390	7.10	4
14	Other allergic agents	Montelukast Sodium 10mg generic	7,208	6.92	29
15	Vitamin B preparations	Mecobalamin 500μg original	6,927	6.65	—
16	Antacids	Magnesium Oxide 330mg generic	6,579	6.32	4
17	Antipyretics and analgesics, anti-inflammatory agents	Loxoprofen Sodium Hydrate 60mg original	6,391	6.14	—
18	Ophthalmic agents	Olopatadine Hydrochloride Solution 0.1% original	6,260	6.01	—
19	Analgesics, anti-itchings, astringents, anti-inflammatory agents	Ketoprofen Tapes 40mg original	6,241	5.99	—
20	Anticoagulants	Heparinoid cream/ lotion/ spray/ gel 0.3% generic	6,130	5.89	14
21	Vitamins A and D preparations	Eldecalcitol 0.75μg original	5,546	5.33	—
22	Antitussives	Dextromethorphan Hydrobromide Hydrate 15mg original	5,530	5.31	—
23	Antidiabetic agents	Sitagliptin Phosphate Hydrate 50mg original	5,426	5.21	—
24	Other digestive organ agents	Dequalinium Chloride 0.25mg generic	5,393	5.18	—

25	Peptic ulcer agents	Vonoprazan Fumarate 10mg original	5,375	5.16	—
26	Gout preparations	Febuxostat 10mg original	5,188	4.98	—
27	Analgesics, anti-itchings, astringents, anti-inflammatory agents	Loxoprofen Sodium Hydrate Tapes 100mg original	5,094	4.89	—
28	Antipyretics and analgesics, anti-inflammatory agents	Acetaminophen 300mg original	5,086	4.89	—
29	Expectorants	L-Carbocisteine 500mg original	4,962	4.77	—
30	Antidiabetic agents	Metformin Hydrochloride 250mg original	4,853	4.66	—
31	Analgesics, anti-itchings, astringents, anti-inflammatory agents	Betamethasone Valerate/Gentamicin Sulfate Ointment 0.12% original	4,825	4.63	—
32	Other allergic agents	Bilastine 20mg original	4,814	4.62	—
33	Antidiarrheals, intestinal regulators	Clostridium butyricum original	4,787	4.60	—
34	Anticoagulants	Heparinoid Ointment 0.3% original	4,768	4.58	—
35	Other allergic agents	Levocetirizine Hydrochloride 5mg original	4,628	4.45	—
36	Ophthalmic agents	Diquafosol Sodium Solution 3% original	4,622	4.44	—
37	Peptic ulcer agents	Rebamipide 100mg original	4,602	4.42	—
38	Antitussives and expectorants	Tipepidine Hibenzate 20mg original	4,536	4.36	—
39	Antipyretics and analgesics, anti-inflammatory agents	Acetaminophen 500mg original	4,515	4.34	—
40	Antacids	Magnesium Oxide 330mg original	4,449	4.27	—
41	Otic and nasal agents	Fluticasone Furoate 27.5μg metered Nasal Spray original	4,227	4.06	—
42	Antitussives	Dihydrocodeine Phosphate/dl-Methylephedrine Hydrochloride/Chlorpheniramine Maleate original	4,209	4.04	—
43	Hyperlipidemia agents	Ezetimibe 10mg original	4,129	3.97	—
44	Analgesics, anti-itchings, astringents, anti-inflammatory agents	Betamethasone Butyrate Propionate Ointment 0.05% original	3,838	3.69	—
45	Peptic ulcer agents	Vonoprazan Fumarate 20mg original	3,662	3.52	—
46	Antidiabetic agents	Linagliptin 5mg original	3,404	3.27	—
47	Other agents affecting central nervous system	Pregabalin 25mg original	3,367	3.23	—
48	Gout preparations	Febuxostat 20mg original	3,362	3.23	—
49	Acting mainly on gram-positive and gram-negative bacteria	Cefcapene Pivoxil Hydrochloride Hydrate 100mg original	3,334	3.20	—
50	Adrenal hormone preparations	Prednisolone 5mg original	3,237	3.11	—

The 50 most-prescribed medications are described according to their therapeutic categories, nonproprietary names, whether they are original or generic products, and their dosage.

Therapeutic categories were allocated according to the package insert or the Prescription Medications in Pharmaceuticals and Medical Devices Agency’s search system [20].
